# Elevated Epithelial Splicing Regulatory Protein 1 Expression in Biliary Atresia Indicates Its Potential as a Molecular Marker

**DOI:** 10.3390/biom16010009

**Published:** 2025-12-19

**Authors:** Giorgia Ammirata, Victor Navarro-Tableros, Marta Manco, Ghania Zubair, Luca Di Costanzo, Luigi Chiusa, Alice Ponte, Michele Pinon, Renato Romagnoli, Ralf Weiskirchen, Paola Cassoni, Pier Luigi Calvo, Ugo Ala, Fiorella Altruda, Sharmila Fagoonee

**Affiliations:** 1 Molecular Biotechnology Center “Guido Tarone”, University of Turin, 10126 Turin, Italy; giorgia.ammirata@unito.it (G.A.); victor.navarro@2i3t.it (V.N.-T.); marta.manco@kuleuven.be (M.M.); luca.dicostanzo@edu.unito.it (L.D.C.); fiorella.altruda@unito.it (F.A.); 2 2i3T-Incubatore di Imprese, University of Turin, 10126 Turin, Italy; 3 Department of Mathematics “Giuseppe Peano”, University of Turin, 10126 Turin, Italy; ghania.zubair@unito.it; 4 Pathology Unit, Department of Medical Sciences, University of Turin and AOU Città Della Salute e Della Scienza, 10126 Turin, Italy; lchiusa@gmail.com (L.C.); paola.cassoni@unito.it (P.C.); 5 Pediatric Gastroenterology Unit, Regina Margherita Children’s Hospital, AOU Città Della Salute e Della Scienza di Torino, 10126 Turin, Italy; alice.ponte@unito.it (A.P.); michele.pinon@gmail.com (M.P.); pierluigi.calvo@unito.it (P.L.C.); 6 Liver Transplant Center, Department of Surgical Sciences, University of Turin and AOU Città Della Salute e Della Scienza, 10126 Turin, Italy; renato.romagnoli@unito.it; 7 Institute of Molecular Pathobiochemistry, Experimental Gene Therapy and Clinical Chemistry (IFMPEGKC), RWTH University Hospital Aachen, D-52074 Aachen, Germany; rweiskirchen@ukaachen.de; 8 Department of Veterinary Sciences, University of Turin, Grugliasco, 10095 Turin, Italy; ugo.ala@unito.it; 9 Institute of Biostructure and Bioimaging (CNR), 10126 Turin, Italy

**Keywords:** ESRP1, hepatobiliary system, cholangiocyte organoids, bioinformatics, cholestasis marker, Biliary Atresia, CFLD, PSC, PBC, mouse models

## Abstract

Cholangiopathies encompass a wide range of chronic liver diseases that target biliary epithelial cells, leading to significant morbidity and mortality due to their progressive nature, limited treatment options, and complex clinical management. Currently, clinically validated biomarkers capable of distinguishing obstructive cholangiopathies, such as biliary atresia (BA), from other cholangiopathies are lacking, hindering timely intervention. RNA-binding proteins (RBPs) have been increasingly linked to human diseases but their roles in cholangiopathies remain underexplored. We assessed the expression of the RBP epithelial splicing regulatory protein 1 (ESRP1) in murine models of cholangiopathies and in the human system. Our findings demonstrate that ESRP1 is highly and specifically expressed in cholestatic liver injury models, including bile duct-ligated, diethoxycarboncyl-1,4-dihydrocollidine-treated, and Mdr2^−/−^ mice when compared with other liver injury models. Importantly, ESRP1 is markedly elevated in the livers of patients with BA and cystic fibrosis-related liver disease, localizing to cholangiocytes and peri-biliary hepatic cells, but is minimal in primary sclerosing cholangitis and primary biliary cholangitis. Moreover, patient-derived BA organoids and biliatresone-treated healthy organoids also display ESRP1 expression. Bioinformatics analysis further implicates ESRP1 in key cholangiopathy-associated pathways, warranting deeper mechanistic investigation. Thus, ESRP1 holds potential as a molecular marker for obstructive cholangiopathies, warranting further mechanistic studies.

## 1. Introduction

Cholangiopathies are a group of chronic, often progressive diseases of the biliary system that can culminate in end-stage liver failure due to limited effective treatments. Biliary atresia (BA), the most common neonatal cholangiopathy, is marked by aggressive inflammation, fibrosis, and obstruction of bile ducts, often requiring liver transplantation if left untreated [1,2]. The pathogenesis of BA is multifactorial and poorly understood, with evidence pointing to genetic predisposition, viral infections, immune-mediated injury, and defects in bile duct remodeling as key contributors. Despite advances in clinical management, the underlying molecular mechanisms driving BA are poorly understood, limiting the development of targeted therapies and improvements in patient outcomes. Recent research emphasizes the importance of exploring not only genetic and transcriptional changes but also post-transcriptional mechanisms, particularly those involving RNA-binding proteins (RBPs) and noncoding RNAs [3,4,5]. Such mechanisms may drive disease progression and offer new therapeutic targets. Moreover, the interaction between RBPs and noncoding RNAs is increasingly recognized as an opportunity for identifying novel molecular markers for human diseases. For example, the long noncoding RNA (lncRNA) MEG9 is significantly upregulated in BA, where it promotes inflammation and fibrosis through potential interaction with S100A9 [3]. MEG9 is predominantly localized in the nucleus of cholangiocytes, where it enhances cell proliferation and migration, as well as the expression of pro-inflammatory cytokines such as CXCL6 and IL6, as well as extracellular matrix components like MMP-7 and collagen I, thereby contributing to liver fibrosis. Circular RNAs (circRNAs) modulate key fibrotic pathways in BA by acting as molecular sponges for microRNAs that regulate transforming growth factor beta (TGF-β) signaling, a central pathway driving hepatic stellate cell activation and fibrosis [4]. MicroRNAs (miRNAs) provide an additional layer of post-transcriptional regulation in BA. MiR-29 family members, particularly miR-29a and miR-29b, are upregulated in experimental BA and regulate key targets such as Igf1, Il1RAP, and DNA methyltransferases (DNMTs), thus influencing fibrosis and inflammatory pathways [6,7,8]. Overexpression of miR-29b in exosomes promotes hepatic stellate cell activation and liver fibrosis by causing hypomethylation and increased expression of platelet-derived growth factor (PDGF) [8]. MiR-29c and miR-145 are downregulated in BA, and their suppression promotes fibrosis by targeting DNMT3A/3B and ADD3, respectively, which are involved in epithelial–mesenchymal transition and fibrogenesis 57 [9,10]. Other miRNAs such as miR-222 and miR-370-3p also modulate hepatic stellate cell proliferation and fibrosis, with miR-222 enhancing fibrosis via Akt signaling and miR-370-3p being regulated by circRNA hsa_circ_0009096 [11,12]. Additionally, circulating miRNAs including miR-140-3p, miR-4429, and miR-4689 show altered expression in BA patients and may serve as potential diagnostic biomarkers [13,14]. Together, these findings highlight how lncRNAs, circRNAs and miRNAs collectively shape the inflammatory and fibrogenic landscape of BA, underscoring their relevance as both mechanistic drivers and potential biomarkers or therapeutic targets. RBPs are also important in various stages of bile acid metabolism, which becomes impaired during cholestasis, and is an initial step in the progression of chronic cholestatic liver disorders [15]. For instance, the RBP ZFP36L1, a downstream target of the central regulator of bile acid balance, Farnesoid X receptor (FXR), has been shown to influence Cytochrome (CYP)-7A1 mRNA stability and regulate bile acid concentrations in rodents through post-transcriptional mechanisms [16]. These findings highlight the importance of further research into the post-transcriptional landscape of BA. Understanding the expression levels and roles of RBPs and their RNA targets could reveal novel biomarkers and therapeutic targets for this devastating disease [17]. Moreover, biliary diseases represent the leading indication for liver transplantation in the pediatric population, and the number of pediatric transplant recipients has remained relatively stable over the past three decades, with BA continuing to be the primary indication. However, the absence of widespread screening programs, combined with persistent diagnostic challenges, has prevented a reduction in the number of children requiring transplantation [18]. Consequently, efforts to improve the differential diagnosis between obstructive biliary diseases and other cholangiopathies, such as progressive familial cholestasis, ductopenia associated with Alagille syndrome, and ciliopathies, are essential. Such efforts are also critical for the identification of potential therapeutic targets.

Epithelial splicing regulatory protein 1 (ESRP1) is a crucial RBP that controls alternative splicing and other post-transcriptional events necessary for maintaining the identity and function of epithelial cells. Its involvement in human diseases, particularly cancer, is intricate and often controversial, as ESRP1 can exert both pro-tumoral and anti-tumoral functions depending on the specific tissue type and disease context [19,20,21]. We and others have shown how aberrant ESRP1 expression is associated with altered cell growth, invasiveness, metastasis, and patient prognosis in colorectal, breast, and ovarian cancers. This is often achieved through its regulation of alternative splicing in genes like the hyaluronic acid receptor CD44 and the tyrosine kinase FGFR2, as well as its impact on the epithelial–mesenchymal transition (EMT) process [21,22,23]. While elevated ESRP1 levels are linked to unfavorable outcomes and enhanced tumorigenic traits in certain cancers, in others, such as lung adenocarcinoma and gastric cancer, ESRP1 seems to hinder invasion and metastasis, demonstrating its dual and context-dependent roles [24,25,26]. Additionally, ESRP1 is essential for maintaining the integrity of the intestinal barrier, and its dysregulation contributes to the development of inflammatory bowel disease [27,28]. Recent studies in intrahepatic cholangiocarcinoma (CCA) indicate that ESRP1 may regulate tumor progression and metastasis through interactions with crucial signaling pathways [29]. Recent studies in intrahepatic CCA indicate that ESRP1 may suppress tumor progression in intrahepatic CCA, for instance, by interacting with ZEB1 and CD44 to regulate EMT [29]. The molecular function of ESRP1 in this cancer type is still under investigation. ESRP1 is also expressed in hepatocellular carcinoma (HCC) where it plays a role in regulating circRNA biogenesis that impacts tumor progression. Specifically, ESRP1 promotes the generation of circPTPN12, a circRNA that suppresses HCC cell proliferation and influences the NF-κB signaling pathway through interaction with PDLIM2, suggesting a tumor-suppressive axis involving ESRP1/circPTPN12/PDLIM2/NF-κB [30]. Additionally, ESRP1 facilitates the biogenesis of circ_0008043, which promotes HCC metastasis by modulating the miR-661/PLEKHG4B axis and EMT, indicating a pro-metastatic role for ESRP1-derived circRNAs in HCC [31]. Although the direct expression levels of ESRP1 in HCC tissues have not been fully characterized, its functional involvement in circRNA regulation and EMT-related pathways highlight its significance in HCC biology. The multifaceted and sometimes contradictory functions of ESRP1 underscore the necessity for further investigation to understand its role beyond cancer in various human diseases, including cholangiopathies [32]. In these conditions, chronic biliary injury and fibrosis involve EMT-like changes regulated by pathways including Hedgehog signaling, which enhances EMT and biliary fibrosis in both human and rodent models [33]. EMT is a prominent feature in BA, and it is closely linked to the progression of liver fibrosis in this disease. Studies show that biliary epithelial cells (BECs) undergo EMT, characterized by the loss of epithelial markers and gain of mesenchymal markers, which contributes to fibrosis development in BA [34,35]. BECs also exhibit a hybrid state with both epithelial and mesenchymal characteristics, with partial EMT playing a critical role in the pathogenesis and fibrosis of BA [36]. Thus, identifying molecular markers capable of detecting a partial EMT state in cholangiopathies is crucial, especially due to its promotion of fibrosis and impaired liver function [35,37]. ESRP1 is a potential candidate marker, and assessing its expression in BA liver tissues could enhance understanding of disease pathogenesis and support the development of diagnostic and therapeutic strategies tailored to pediatric cholangiopathies.

In this study, we demonstrate the overexpression of ESRP1 in murine models of cholangiopathies, as well as in the livers of patients with BA and biliatresone (BLZ)-treated cholangiocyte organoids (CHOs). This RBP is not expressed in healthy livers. This suggests that ESRP1 could serve as a potential molecular marker and a novel player in the pathogenesis of BA, the most common cause of infantile cholestasis requiring liver transplantation, which is often diagnosed by exclusion after ruling out other specific disorders. Additionally, ESRP1 exhibits increased expression in cystic fibrosis-related liver disease (CFLD) livers, but not in primary sclerosing cholangitis (PSC) or primary biliary cholangitis (PBC), indicating the need for further mechanistic investigations.

## 2. Materials and Methods

### 2.1. Patients Characteristics and Ethics Statement

Samples used for this study were collected at Regina Margherita Children’s Hospital in Turin. Liver biopsies were performed at the time of Kasai portoenterostomy (KPE), after approval by the A.O.U. Città della Salute e della Scienza di Torino Institutional Ethics committees (n. 0002317 on 26 May 2025, entitled “New molecular therapies and Biomarkers for pediatric cholangiopathies”). Since all the patients participating in this study were minors, written informed consent was obtained from their parent/guardian at the time of sample collection after explaining the objectives and outcomes of the study.

### 2.2. Livers of Mice with Hepatobiliary Injury

Frozen livers for gene expression analysis were obtained from previously generated mouse models of hepatobiliary injury, including bile duct ligation (BDL), diethoxycarboncyl-1,4-dihydrocollidine (DDC) treatment, *Mdr2* deficiency and carbon tetrachloride (CCl_4_) treatment [38]. Partial hepatectomy (PHx) was performed as previously described, with 70% of the liver removed [39]. For the analysis of fresh hepatocytes, 8-week-old C57BL/6J male mice were subjected to either BDL or a sham operation (sham) for 1, 4, 8 and 15 days as previously described [38]. Gall bladders were collected and washed before being snap-frozen. Hepatocytes were isolated from livers following a previously described protocol [40]. Briefly, after liver perfusion with liver digest medium (Thermofisher Scientific, Milan, Italy) at 37 °C, isolated cells were passed through a 70 μm strainer, pelleted via low-speed centrifugation, and then frozen for further analysis. Intrahepatic bile ducts that were separated during this procedure were also frozen for gene expression analysis. All experiments were performed in accordance with the Italian legislation on protection of animals (Protocol number: CC652.109) and the University of Turin Guidelines.

### 2.3. RNA Extraction and Gene Expression Analysis

Total RNA was extracted from the tissue samples collected from BA patients using Purelink^TM^ RNA Mini kit (Life Technologies, Milan, Italy) according to the manufacturer’s instructions and quantified using Nanodrop system (Life Technologies, Milan, Italy). RNA extraction from mice liver samples and cholangiocyte organoids (CHOs) was performed using TRIzol™ Reagent (Thermo Fisher Scientific, Milan, Italy). For quantitative gene expression analysis, we used the primers listed in Table S1 and ran them on a Quantstudio Flex 6 real-time PCR systems. Taqman custom gene expression assay panels were also used for some experiments (ESRP1 and 18S; Thermo Fisher Scientific, Milan, Italy). Data were analyzed using the ΔΔCt method. Transcript abundance, normalized to 18S or GAPDH mRNA expression, was expressed as a fold change relative to a calibrator sample.

### 2.4. Western Blot Analysis, Histological Analyses and Immunohistochemistry

Protein extracts were prepared from liver tissues using RIPA buffer supplemented with protease and phosphatases inhibitors, as well as PMSF (Sigma-Aldrich, Milan, Italy). SDS-PAGE (Bio-Rad, Segrate, MI, Italy) was performed as previously described using anti-ESRP1 (HPA023719, Clinisciences, Nanterre, France) or anti-vinculin (lab-made, Molecular Biotechnology Center, Turin, Italy) antibodies [22]. Formalin-fixed, paraffin-embedded liver tissues were cut into 5 µm sections. Collagen deposition was assessed by Picrosirius Red staining. For immunohistochemistry, sections were stained with anti-ESRP1 (Clinisciences, Nanterre, France) and anti-cytokeratin (CK)-7 (Ventana Medical System, Inc., Tucson, AZ, USA) antibodies following standard protocols [26]. For hematoxylin and eosin (H&E) staining, CHO samples were embedded in Tissue-Tek-II (Bio-optica, Milan, Italy) and frozen at −80 °C. Cryostat sections (10 μm) were serially prepared and fixed in acetone for 5 min, then routinely stained with hematoxylin and eosin (H&E, Merck, Milan, Italy) for microscopic evaluation.

### 2.5. Cholangiocyte Organoid Generation

CHOs were generated as previously described, with some modifications [41]. Briefly, patient tissues (liver or extrahepatic tissues) were immersed in NaCl 0.9%, transported to the laboratory at 4 °C, and processed within 30 min after resection. After washing thoroughly with PBS to eliminate bile, tissues were mechanically cut into small pieces (<1 mm) using a scalpel, followed by washing with PBS containing 1% BSA (*W*/*V*) and pelleting at 400 g (1500 rpm), for 5 min at 4 °C. The pellet was mixed in an ice-cold Geltrex-based solution (Thermo Fisher, Milan, Italy) consisting of 66% Geltrex and 34% organoid expansion medium, then plated as 50 µL Geltrex-medium domes in 10 cm Petri dishes (Sardsted, Milan, Italy) and left to polymerize before culturing in expansion medium. The expansion medium contained DMEM/F12 supplemented with 2 mM Glutamine, Penicillin/Streptomycin (100 U/mL and 100 µg/mL, respectively), 1% Neuro-2 medium supplement, 1% B-27 supplement, 1.25 mM N-Acetylcysteine, 10 nM Gastrin, 50 ng/mL EGF, 100 ng/mL FGF10, 25 ng/mL HGF, 500 ng/mL R-spondin, 10 mM Nicotinamide, 5 µM A 83-01, 2 µM Forskolin, 25 ng/mL Noggin, 10 µg/mL Wnt3, and 10 µM Y27632 (all from Thermo Fisher, Milan, Italy).

#### 2.5.1. Biliatresone-Induced Injury

BLZ-induced injury was achieved by cultivating CHOs in expansion medium supplemented with 2 μM or 20 μM of BLZ (Merck-Sigma, Milan, Italy) for 24 h, 48 h or 144 h, without replacing the medium [42].

#### 2.5.2. Rhodamine 123 Assay

The Rhodamine 123 (Rho123) assay, a fluorescence-based technique for assessing membrane transport activity, was used to evaluate the secretory function of CHOs. In this procedure, CHOs were incubated at 37 °C for 30 min in expansion medium alone or in presence of verapamil, secretin, or somatostatin (10 µM; Sigma-Aldrich, Milan, Italy). After incubation, the organoids were washed twice with PBS and then cultured in CHO expansion medium containing 100 µM Rho123 at 37 °C for 30 min (Sigma-Aldrich, Milan, Italy). The organoids were washed in PBS and maintained in fresh CHO expansion medium until analysis. Intracellular Rho123 accumulation was assessed by fluorescence microscopy (Leica Microsystems, Milan, Italy). For each treatment condition, representative micrographs were acquired, and fluorescence intensity distribution profiles were quantified using ImageJ software (version 2.1, https://imagej.net/ij/; accessed on 8 May 2025).

### 2.6. Bioinformatics and Statistical Analyses

#### 2.6.1. Data Retrieval and Bioinformatics Analysis

Data for PSC, PBC, and CCA were obtained from CHOL TCGA (https://xenabrowser.net/datapages/, last accessed on 15 October 2025), and from the NCBI Gene Expression Omnibus (GEO): GSE119600, GSE144521, GSE159676. The expression of *ESRP1* and other genes were evaluated, in Gene Expression Omnibus (GEO) public datasets, with Mann–Whitney U test or Kruskal–Wallis test, with subsequent pairwise comparisons with FDR correction (where appropriate). In CHOL TCGA dataset, gene expression was assessed through EBSeq library in R [15]. The bulk RNA-seq dataset used in this study was obtained from the NCBI GEO with the accession number GSE122340 [15,43]. It comprised 178 samples, including 171 from patients diagnosed with BA. Based on *ESRP1* gene expression levels, the samples were stratified into three equally sized groups, designated as “high”, “medium”, and “low” expression categories. The same dataset contained 7 healthy individuals; however, one of these displayed an outlier-like expression profile related to *ESRP1*, showing a gene expression pattern markedly different from the other normal samples and was thus discarded. In the current study, only subjects belonging to the ESRP1^high^ and ESRP1^low^ subgroups were further processed. Pearson correlation coefficients were calculated using the cor.test function from the stats package. Differential gene expression analysis was performed with DESeq2 (version 1.48.2), following its standard workflow: estimation of dispersion, fitting of the negative binomial model, and application of Wald tests. Condition was used as the design factor to construct a DESeq2 dataset after low-abundance genes (less than 10 total reads) were removed. Functional enrichment analyses were carried out using the clusterProfiler package (version 4.16.0). Gene Ontology (GO) enrichment was assessed separately for the Biological Process (BP), Molecular Function (MF), and Cellular Component (CC) categories, while Kyoto Encyclopedia of Genes and Genomes (KEGG) pathway analysis was performed for *Homo sapiens*. For correlation, differential expression and enrichment analyses, statistical significance was determined by the False Discovery Rate (FDR) using Benjamini–Hochberg-adjusted *p*-values. All statistical analyses were conducted in R (version 4.5.1).

#### 2.6.2. Statistical Analysis

Data from experimental groups were compared using one-way ANOVA with Bonferroni post hoc testing in GraphPad Prism 5. Statistical significance was defined as * *p* < 0.05, ** *p* < 0.01, and *** *p* < 0.001.

## 3. Results

### 3.1. ESRP1 Is Expressed in Murine Livers with Chronic Biliary Injury

**Figure 1:**
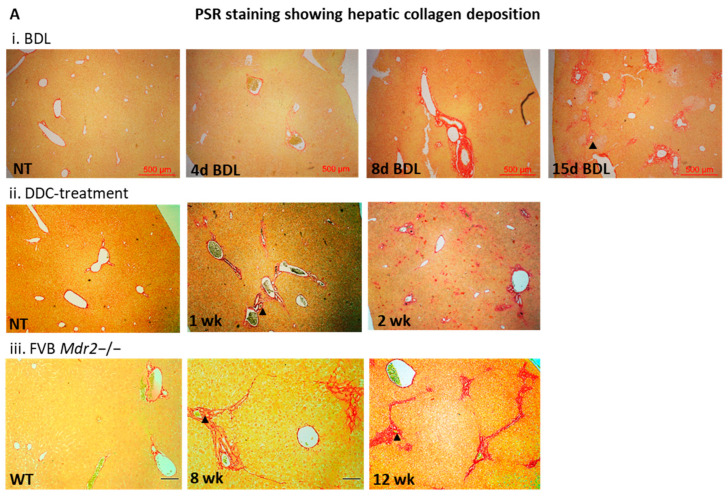

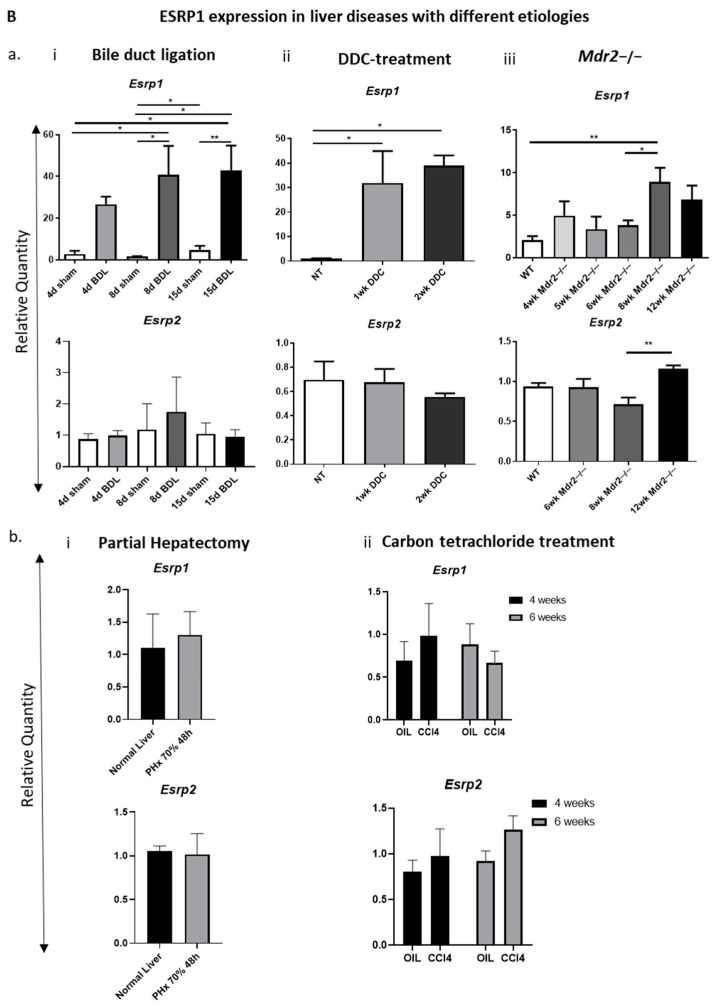
(**A**). Liver histology of cholestatic mice stained with Picrosirius Red (PSR) (**i**) BDL, (**ii**) DDC diet, (**iii**) *Mdr2*^−/−^ at 4× magnification (arrowheads show ductular reaction). (**B**) Etiology-dependent ESRP1 expression in murine liver. Quantitative (q)-RT-PCR analysis of Esrp1 and Esrp2 expression, normalized to 18SrRNA expression, in livers from (**a**). (**i**) BDL, (**ii**) DDC-fed and (**iii**) *Mdr2*^−/−^ mice, compared to those of (**b**). (**i**) 70% partial hepatectomy or (**ii**) CCl_4_-treated mice (*n* = 6/group). Statistical significance is indicated as * *p* < 0.05, ** *p* < 0.01.

The development of fibrosis was monitored in all mouse models (BDL, DDC-diet and Mdr2^−/−^) by Picrosirius red staining of liver sections, showing a gradual increase in fibrosis over time. Notably, liver fibrosis was clearly evident after 8 days of BDL, 1 week of DDC feeding, and in 4-week-old *Mdr2*^−/−^ mice (Figure 1A), as well as after 4 weeks of CCl_4_ treatment, as we previously reported [38].

Analysis of biochemical parameters and H/E staining showed results consistent with previous reports [38]. PHx (70%) did not induce liver fibrosis.

**Figure 2:**
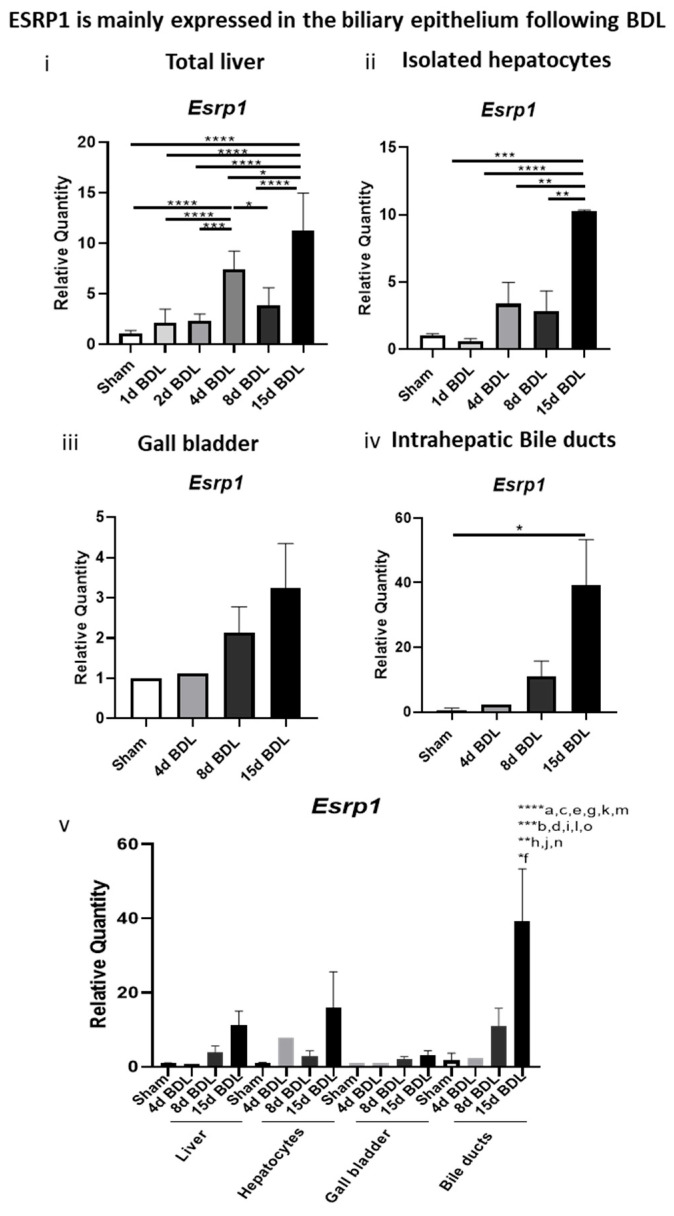
ESRP1 expression in various hepatobiliary compartments of BDL livers. RT-qPCR analysis of ESRP1 gene expression was conducted in the same setting. “Bile ducts” are intrahepatic biliary tissue. Statistical significance is indicated as * *p* < 0.05, ** *p* < 0.01, *** *p* < 0.001, **** *p* < 0.0001; 15d BDL versus a–o where the latter represents the other conditions ranging from Sham to 8d BDL, respectively.

We investigated the expression of the RBP ESRP1 in the livers following injury at multiple experimental time points. Compared with livers from mice subjected to 70% PHx or CCl_4_ administration, livers from mice with chronic cholestasis exhibited markedly higher *ESRP1* expression. Notably, *ESRP1* upregulation was evident as early as day 4 after BDL, suggesting that early cholangiopathic events drive *ESRP1* induction prior to the onset of histologically detectable fibrosis (Figure 1B). *ESRP1* expression also increased after 2 weeks of DDC-diet feeding and remained elevated until 8 weeks (the last time point analyzed) (Figure S1). On the other hand, the expression of the paralog *ESRP2,* which is highly expressed in the liver and is responsible for maintaining the mature phenotype of adult hepatocytes, remained constant during the development of hepatobiliary injury (Figure 1B) [44]. To determine the cellular sources of *ESRP1*, we isolated hepatocytes from BDL livers and examined intrahepatic bile ducts as well as the gallbladder, an extrahepatic component of the biliary system. After 15 days of BDL, *ESRP1* expression was significantly elevated in both hepatocytes and intrahepatic bile ducts (Figure 2). DDC-exposed hepatocytes also expressed *ESRP1* with respect to controls, while *ESRP2* expression showed no change (Figure S1).

Importantly, the intrahepatic biliary epithelium expressed the highest level of ESRP1 compared to the hepatocytes and gall bladder. This finding prompted us to further investigate ESRP1expression in human cholangiopathies.

### 3.2. ESRP1 Is Highly Expressed in Livers of BA Patients

**Figure 3:**
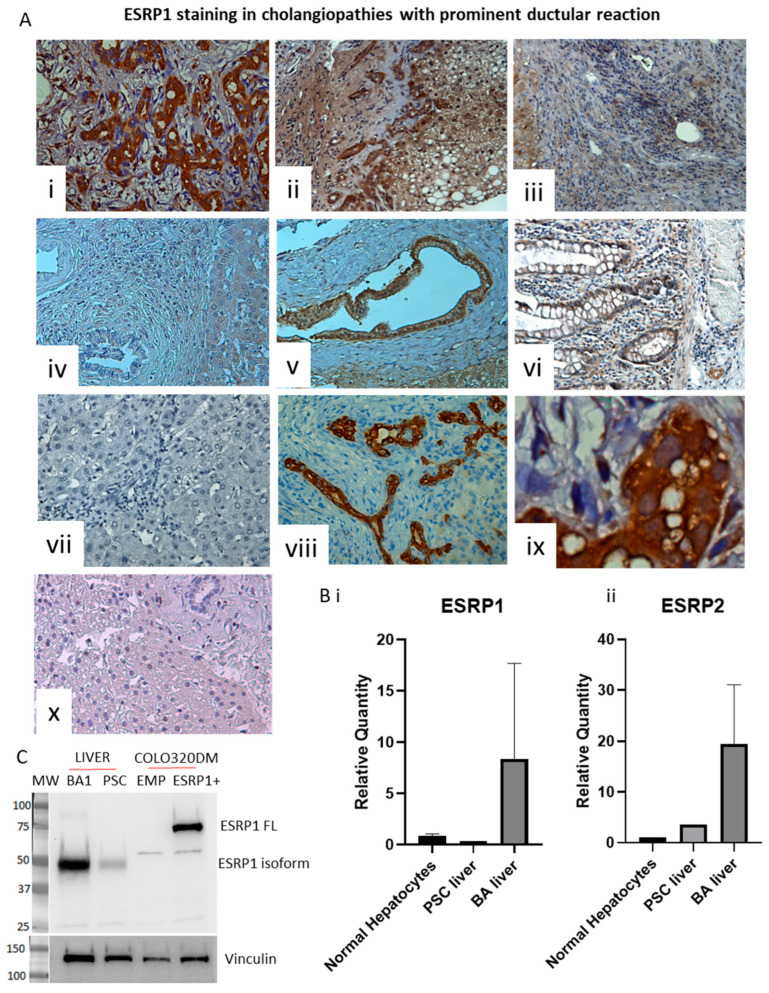
(**A**) Immunohistochemical staining for ESRP1 in liver biopsy sections from patients with cholestatic diseases. (**i**) Biliary atresia (BA) (**ii**) Cystic fibrosis-related liver disease (CFLD); (**iii**) primary biliary cirrhosis (PBC); (**iv**) primary sclerosing cholangitis (PSC); (**v**) intrahepatic bile duct. Colon mucosa (**vi**) was used as a positive control; liver tissue without the primary antibody (**vii**) was used as a negative control. (**viii**) Representative CK7-positivity is shown. Images were taken with a BX41 Olympus microscope at 20× magnification. Of note, ESRP1 staining is both nuclear and cytoplasmic in positive cells (**ix**). (**x**) Normal liver stained for ESRP1. (**B**). Quantitative real-time PCR analysis of (**i**) ESRP1 and (**ii**) ESRP2 expression in normal isolated hepatocytes (no normal liver biopsy was available) versus PSC and BA whole livers. (**C**) Western blot analysis of ESRP1 expression in BA and PSC livers; a representative BA sample was analyzed. Colo320DM cells expressing ESRP1 were used as positive control; MW: molecular weight. Original Western blot images can be found in Figure S9.

To confirm the preclinical data, paraffin-embedded liver sections from patients with BA, PSC, PBC and CFLD were obtained from the Pathological Anatomy Department of AOU Città della Salute e della Scienza, Turin, Italy. Analysis of liver sections from cholestatic patients showed high ESRP1 expression in the intrahepatic biliary epithelium as well as in surrounding hepatocytes (Figure 3).

Interestingly, a shorter isoform of ESRP1 was detected in human livers in comparison to the full-length version previously identified in colorectal cancer cell lines (Figure 3C) [26]. ESRP1 expression correlates with the presence of a prominent ductular reaction in conditions such as BA and CFLD. It is also found in surrounding hepatocytes, which may represent hepatic progenitor cells that expand during liver injury. In contrast, both PSC and PBC livers do not show discernible ESRP1 expression (Figure 3A). As expected, ESRP1 is not expressed in normal hepatocytes or liver tissue. These findings were confirmed by gene expression analysis (Figure 3B).

### 3.3. Generation of Cholangiocyte Organoids from Livers of BA Patients

**Figure 4:**
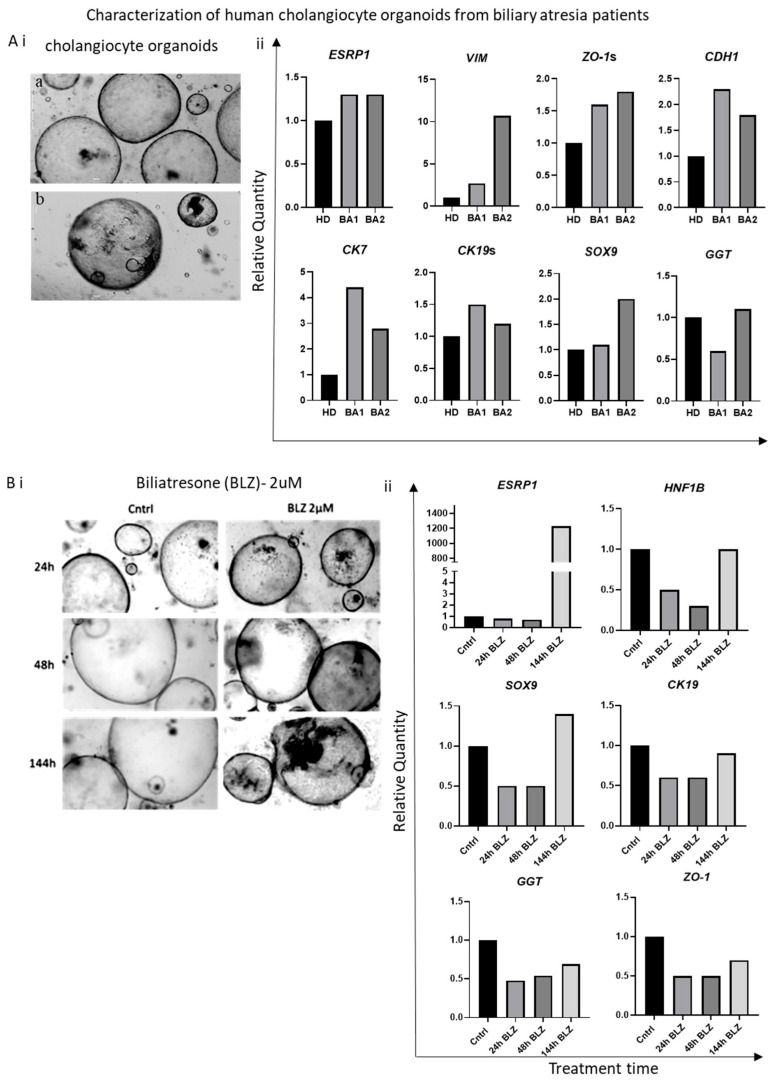
Characterization of human CHOs derived from a healthy donor (HD) or patients with BA (BA1 and BA2). (**A**). (**i**) Bright-field representative images displaying morphological differences between representative healthy (H)-CHOs (**a**) and Biliary Atresia (BA)-CHOs (**b**). Scale bar = 1000 µm. (**ii**) Molecular profile of H-CHOs and BA-CHOs showing the expression of ESRP1, CDH1 (epithelial genes), VIMENTIN (mesenchymal gene), SOX9, CK7, CK19, (cholangiocyte markers), ZO-1 (tight junction marker) and GGT (cholestatic marker). (**B**) In vitro BLZ-induced BA model in CHOs. (**i**) Representative micrographs of DMSO-treated control CHOs (Cntrl) and those treated with a single dose of 2 µM BLZ. (**ii**) Analysis of ESRP1 (epithelial RBP), SOX9, CK19, HNF1B (cholangiocyte markers), ZO-1 (tight junction marker) and GGT (cholestatic marker) expression in BLZ-treated CHOs at 24 h, 48 h and 144 h, compared to DMSO-treated controls (Cntrl).

Human primary cholangiocytes were isolated from fresh liver biopsies or from extrahepatic biliary tissue obtained from healthy donors or two BA patients. The cells were used for molecular characterization and CHO generation (Table 1, Figure 4A and Figure S2).

Healthy (H)-CHOs displayed a typical cystic phenotype, characterized by a regular spheroidal morphology with cholangiocytes aligned along the outer layer surrounding a fluid-filled core (Figure 4A and Figure S3). In contrast, Biliary Atresia (BA)-CHOs exhibited morphological variations resembling the clinical conditions of the donors. Specifically, BA-CHOs showed discontinuities (holes; white arrowheads) in the outer capsule compared to the intact morphology of H-CHOs. Rho123 fluorescence assay revealed higher basal accumulation of this substrate within the lumen of BA-CHOs compared to H-CHOs (Figure S3). This uptake was markedly reduced in both CHO types following Verapamil treatment, indicating preservation of the calcium-dependent modulation. Interestingly, Secretin treatment resulted in a decreased Rho123 uptake in BA-CHOs relative to H-CHOs. The response of BA-CHOs to Somatostatin treatment was also altered, characterized by a reduction in Rho123 transport. Notably, *ESRP1* expression exhibited an increasing trend in BA-CHOs relative to H-CHOs (Figure 4(Aii)). Molecular analysis confirmed the cholangiocyte identity, while the upregulation of EMT-associated genes such as *VIMENTIN* and *CDH1* (E-CADHERIN) indicated that BA-CHOs possess partial EMT characteristics (Figure 4(Aii) and Figure S2).

To determine whether *ESRP1* expression is induced during the onset of BA, H-CHOs were treated with BLZ, an isoflavonoid known to target extrahepatic cholangiocytes and induce BA-like pathology [45]. Two concentrations of BLZ (2 μM and 20 μM) were tested. However, the higher concentration caused severe cytotoxicity and was therefore excluded from further analysis (Figure S4). H-CHOs treated with 2 μM BLZ were analyzed at various time points over 144 h (Figure 4B). BLZ treatment induced BA-like features in the CHOs, resembling those observed in BA-CHOs (Figure 4A), most prominently at 144 h (Figure 4(Bi)). While the expression of cholangiocyte markers remained stable, *ESRP1* expression was markedly upregulated only at 144 h post-treatment, coinciding with the emergence of BA-like morphological features (Figure 4(Bii)).

### 3.4. Bioinformatics Input into ESRP1 Expression and Gene Network in a Large Human BA Cohort

**Figure 5:**
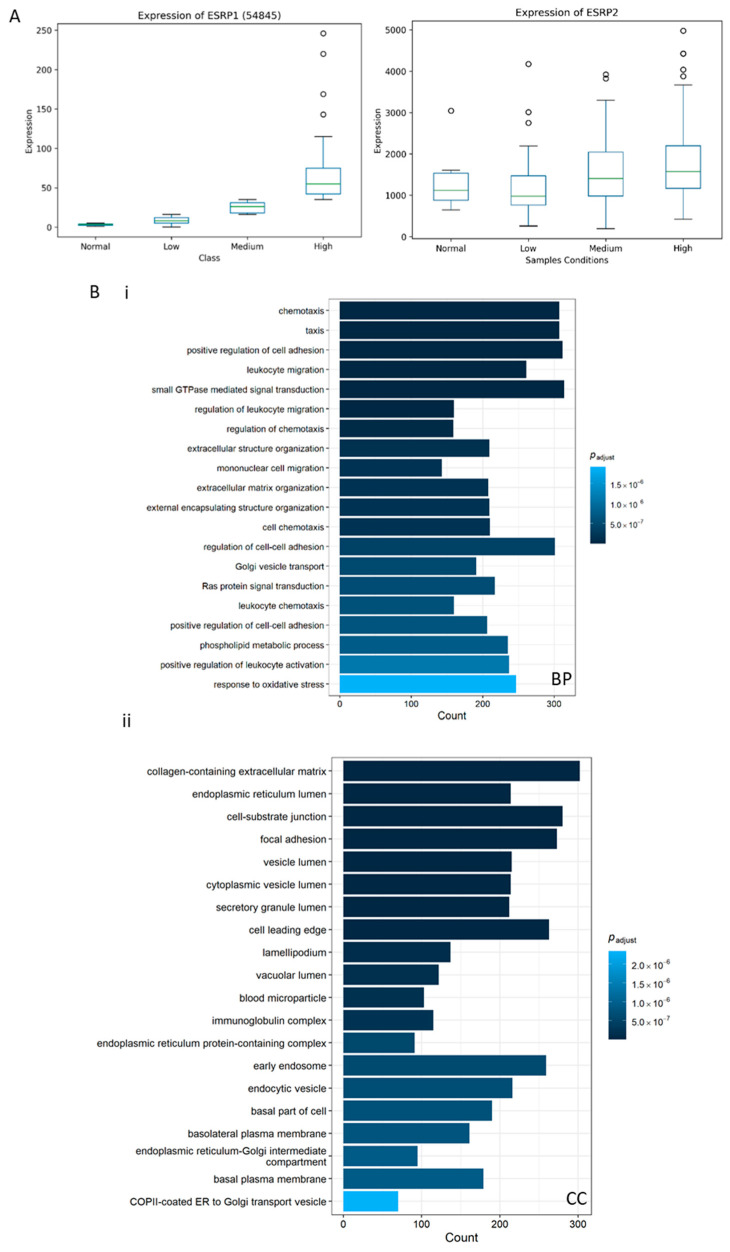

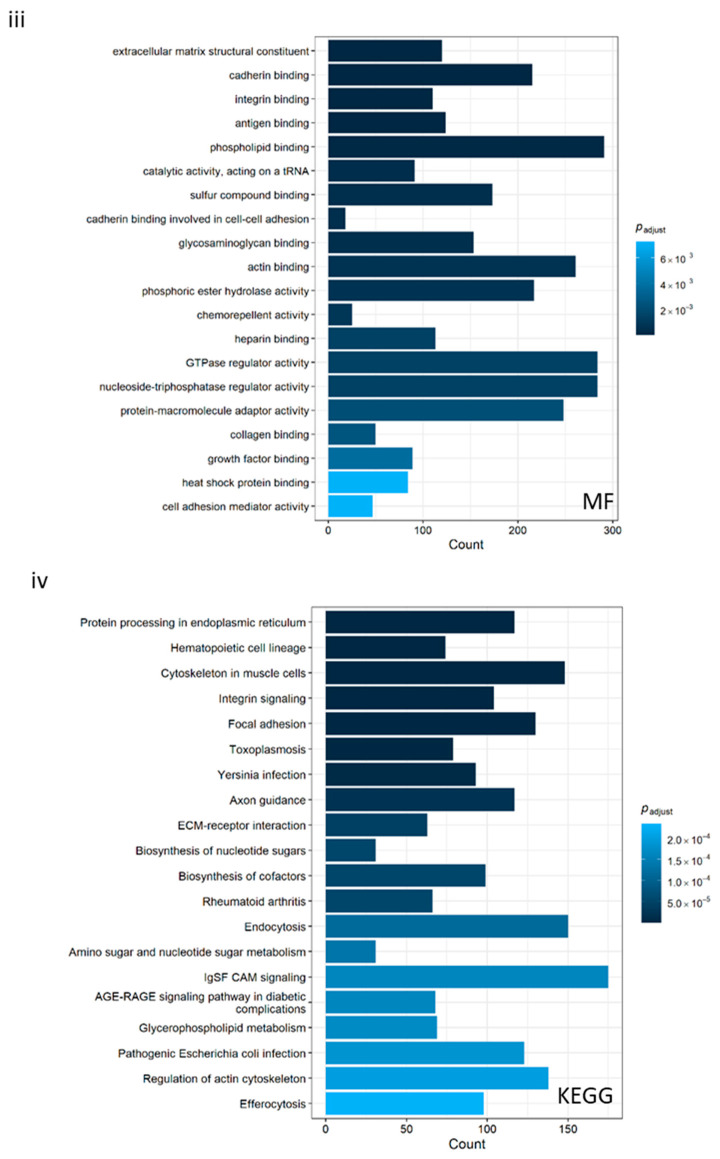
Bioinformatics analysis of ESRP1 expression and function. (**A**) ESRP1 and ESRP2 expression levels in BA patients (*n* = 171) compared with normal controls (*n* = 6) from the GSE122340 dataset. BA patients were stratified based on ESRP1 expression into three groups: ESRP1^high^, ESRP1^medium^ and ESRP1^low^ (comparable to normal). (**B**) GO analysis of differentially expressed genes (DEGs) in the ESRP1^high^ group with respect to normal subjects. The top enriched categories for (**i**) Biological Process (BP), (**ii**) Cellular Component (CC), (**iii**) Molecular Function (MF), and (**iv**) KEGG pathway analysis are shown.

To corroborate our data on ESRP1 expression in liver sections from cholangiopathic patients (Figure 3), we analyzed several publicly available datasets related to BA, PSC, PBC, and CCA for *ESRP1* expression [15]. Notably, livers from BA patients exhibited markedly higher levels of ESRP1 expression compared to normal tissues, consistent with our experimental observations (Figure 5A).

Data from the CHOL TCGA dataset revealed that *ESRP1* expression was significantly upregulated in CCA tumor tissues compared with their respective normal samples (fold change = 2.008; *p* = 1.57 × 10^−6^) (Figure S5). Additional transcriptomic datasets were retrieved from GEO. The GSE119600 dataset includes whole-blood samples from patients with PBC and PSC, as well as healthy controls [46]. In this dataset, *ESRP1* expression did not differ significantly among the groups (*p* = 0.2862) (Figure S5). The GSE144521 dataset comprises serum and urine extracellular vesicle (EV) profiles from patients with PSC, CCA, and healthy subjects, together with data from normal (NHC_WCE), immortalized (H69_WCE), and tumor-derived (EGI_WCE, TFK_WCE) cholangiocyte cell lines [47]. Although *ESRP1* expression in serum and urine EVs showed no significant differences (*p* = 0.1088 and *p* = 0.6653, respectively), analysis of the cholangiocyte cell lines revealed markedly higher *ESRP1* expression in tumor-derived cells compared with normal and immortalized cholangiocytes (*p* = 0.01338) (Figure S5). The GSE159676 dataset includes frozen liver tissue samples from healthy controls (Normal), PSC, and PBC patients, with no significant differential expression of *ESRP1* observed among the groups (*p* = 0.6451) [48].

We used BA livers for successive ESRP1 expression and correlation analyses. To identify new transcripts that may be involved in ESRP1-mediated regulatory mechanisms in BA, we established a multi-step analytical pipeline based on the GSE122340 dataset.

#### 3.4.1. Analysis of Differential Expression

Initially, we conducted differential expression (DE) analyses to identify genes that showed significant expression changes across the dataset. Notably, ESRP1 exhibited a significant upregulation not only in the ESRP1^high^ group (log_2_FC = 3.46, adjusted *p* = 2.04 × 10^−13^), but also in the ESRP1^low^ group when compared with normal samples (log_2_FC = 1.32, adjusted *p* = 0.015). DE was specifically conducted between ESRP1^high^ samples and normal subjects, as well as between ESRP1^low^ samples and normal subjects (Figure S6).

#### 3.4.2. Functional Enrichment and Pathway Analysis in ESRP1^high^ BA

To elucidate the biological processes associated with genes modulated similarly to ESRP1, all statistically differentially expressed genes in the ESRP1^high^ subgroup, without applying fold-change thresholds, were analyzed for functional enrichment across GO and KEGG categories (Figure 5B and Table S2). The resulting enrichment patterns indicated predominant activation of inflammatory, immune, and metabolic pathways, consistent with a highly reactive, injury-associated hepatic microenvironment.

In the GO BP category, the most significantly enriched terms included chemotaxis, leukocyte migration, positive regulation of cell adhesion, cell–substrate adhesion, and regulation of chemotaxis, highlighting extensive immune cell recruitment and adhesion remodeling. Enrichment in response to oxidative stress, response to endoplasmic reticulum stress, and wound healing reflects the stress-adaptive and reparative responses of hepatobiliary tissue under inflammatory pressure. Processes such as glycerophospholipid metabolism and biosynthesis, together with lipid catabolic pathways, indicate concurrent metabolic remodeling and altered membrane dynamics. The CC ontology reinforced these observations, showing enrichment in extracellular matrix structures, focal adhesion, basement membrane, endoplasmic reticulum, lysosome, and secretory granule structures, consistent with matrix remodeling, protein trafficking, and inflammatory secretion. At the MF level, enriched terms such as integrin binding, cadherin binding, collagen binding, actin binding, metallopeptidase activity, and chemokine activity suggest extensive intercellular adhesion and extracellular matrix degradation, hallmarks of tissue inflammation and fibrogenesis. Corresponding KEGG pathways included cytokine-cytokine receptor interaction, chemokine signaling, NF-*κ*B signaling, T cell receptor signaling, and Th1, Th2 and Th17 differentiation, confirming strong engagement of immune activation networks. Additional enrichment in ECM–receptor interaction, PI3K-Akt, MAPK, TGF-β, and Hedgehog signaling pathways indicates parallel activation of fibrogenic and pro-inflammatory cascades, while bile secretion, fatty acid metabolism, and arachidonic acid metabolism highlight hepatic metabolic disturbance.

Overall, the ESRP1^high^ subgroup transcriptional profile in BA reflects a pro-inflammatory and remodeling-prone state, characterized by immune infiltration, oxidative stress, extracellular matrix reorganization, and metabolic adaptation.

#### 3.4.3. Correlation-Based Analyses

**Figure 6:**
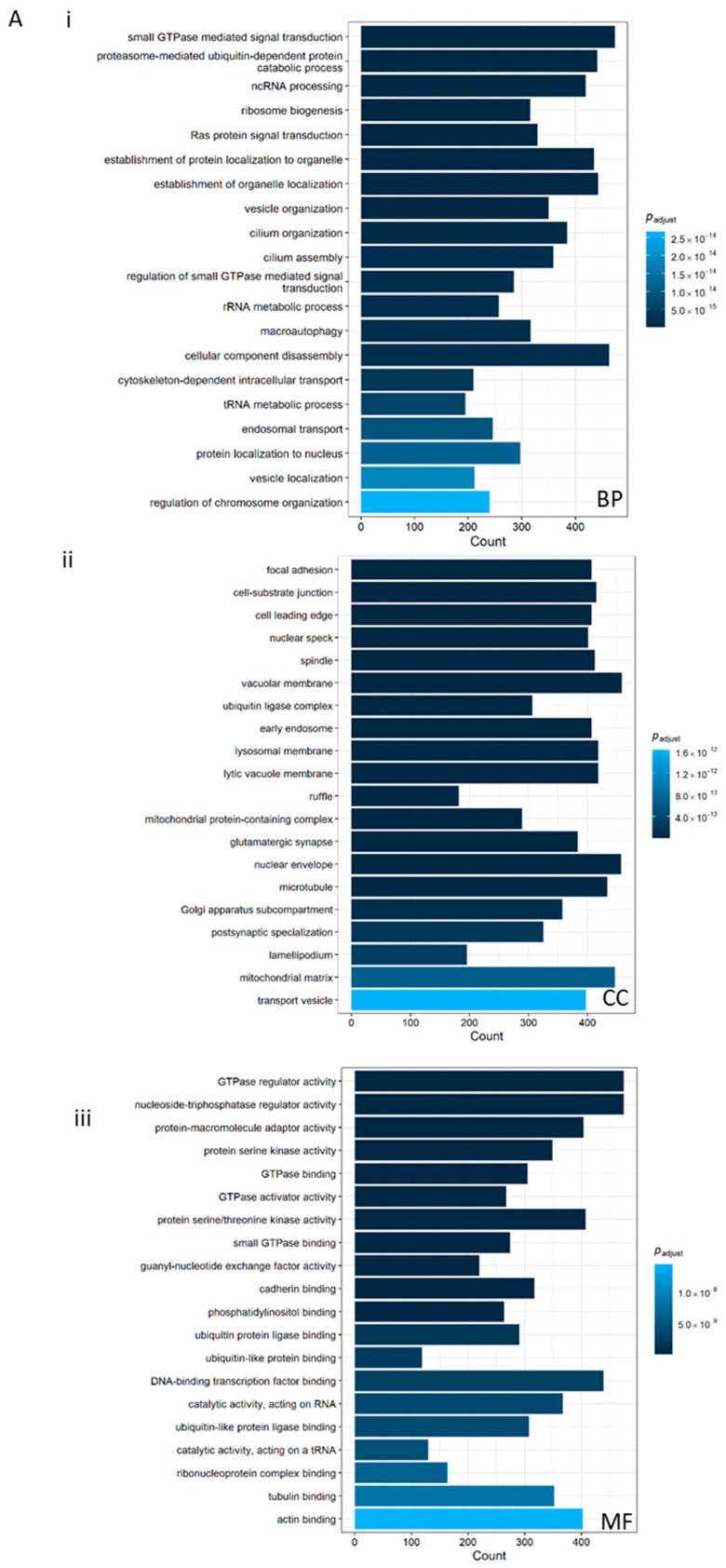

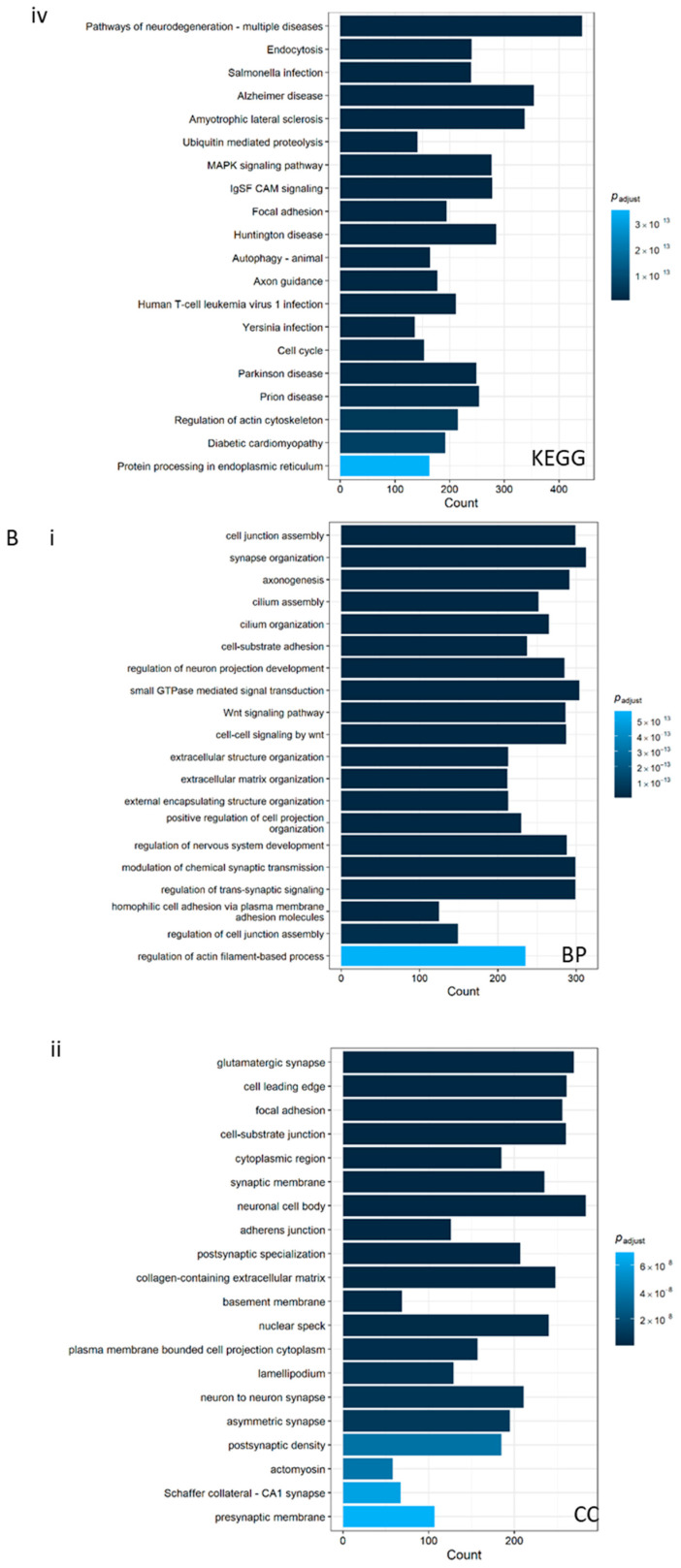

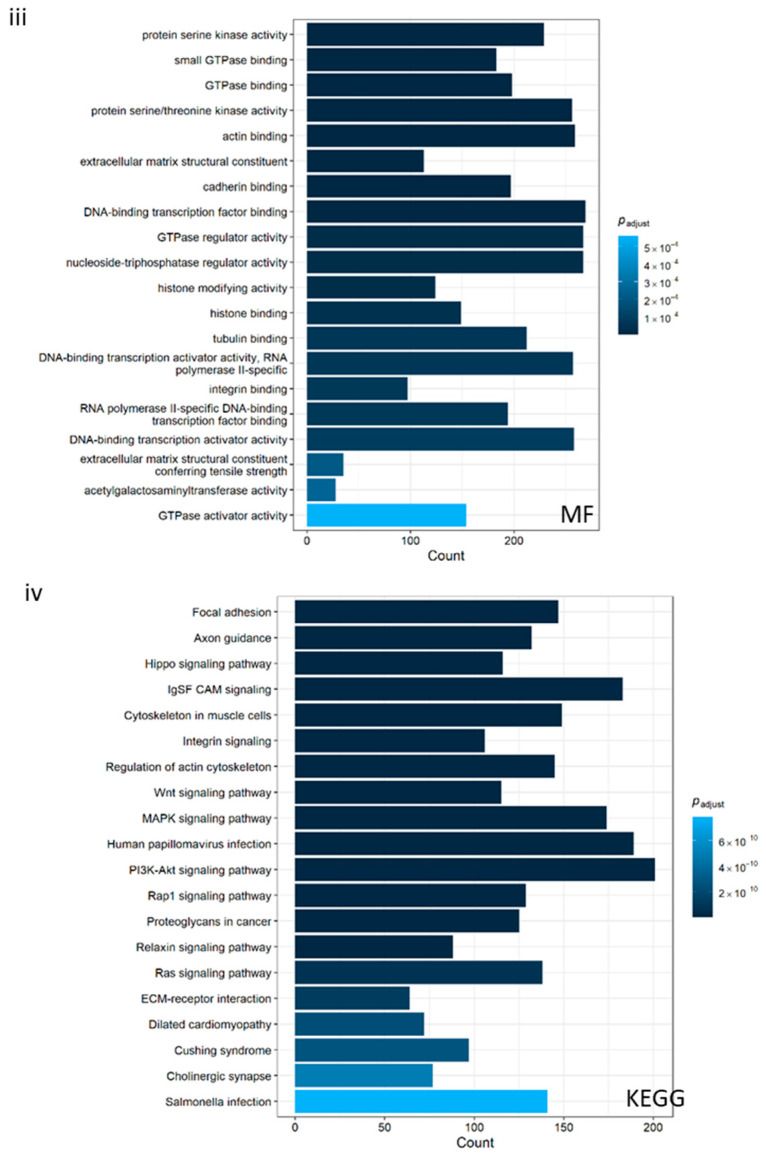
Bioinformatics analysis of ESRP1-correlated gene expression in BA. GO of genes positively correlated with ESRP1 in (**A**) all BA patients and (**B**) ESRP1^high^ BA patients. The top enriched categories for (**i**) Biological Process (BP), (**ii**) Cellular Component (CC) and (**iii**) Molecular Function (MF) and (**iv**) KEGG pathway analysis are displayed.

Subsequently, correlation-based analyses were applied as an additional filtering layer to refine the candidate gene set. Pearson correlation coefficients were calculated to identify genes whose expression profiles were significantly positively associated with *ESRP1* both across the entire BA cohort and within each expression-defined subgroup (Figure 6 and Figure S7, and Table S2).

This dual-level approach allowed for the identification of genes that exhibit co-expression patterns suggestive of shared regulatory control or functional interdependence with *ESRP1*.

#### 3.4.4. Shared and Specific Functional Characterization of ESRP1-Correlated Genes

Comparison of the enrichment profiles from all BA samples and the ESRP1^high^ subgroup revealed significant overlap in cytoskeletal, adhesion, and signaling processes, along with distinct functional signatures suggesting enhanced epithelial organization and remodeling in the ESRP1^high^ condition.

Both datasets shared enrichment for pathways regulating cell–matrix adhesion, actin cytoskeleton dynamics, and vesicle-mediated transport, including focal adhesion, cell junction assembly, cell–substrate adhesion, microtubule-based transport, and small GTPase- and Ras-mediated signal transduction. Common KEGG pathways such as *PI3K-*Akt, MAPK, Wnt, and Ras signaling, along with Hippo signaling, underscore a conserved network of epithelial maintenance, growth, and fibrogenic signaling central to biliary tissue remodeling. Both contexts also exhibited enrichment in the endoplasmic reticulum context.

Distinctive features of the ESRP1^high^ subgroup included stronger enrichment for extracellular matrix organization, epithelial tube morphogenesis, actomyosin and focal adhesion assembly, EMT, and response to TGF-β, suggesting enhanced coordination of junctional remodeling and epithelial polarity restoration. These processes are critical for maintaining bile duct integrity and regulating reparative fibrosis. Enrichment in integrin binding and collagen organization further emphasized a shift toward structural stabilization and tissue regeneration rather than inflammation-driven injury.

Taken together, these findings indicate that while ESRP1-associated pathways in BA broadly govern adhesion, cytoskeletal organization, and signaling cross-talk, the ESRP1^high^ subset displays a transcriptional bias toward epithelial reconstruction and extracellular matrix reorganization, consistent with a role for ESRP1 in promoting ductal epithelial resilience and controlled fibrogenic remodeling within the injured liver.

#### 3.4.5. Enhanced Identification of Putative ESRP1-Related Genes

To refine the identification of putative ESRP1-related genes, we implemented a stricter overlap strategy. We included genes that (i) showed significant correlation with ESRP1 across the entire BA cohort as well as within both ESRP1-defined groups (ESRP1^high^ and ESRP1^low^) and (ii) were up-regulated in ESRP1^high^ compared to ESRP1^low^ (log2FC > 1). This selection process resulted in a total of 282 transcripts that displayed consistent, ESRP1-concordant directional changes across the subgroups.

To better understand the relevance of these potential genes in biliary epithelium biology, we compared the 282-gene set with the Cholangiocyte Gene Set (Harmonizome 3.0; https://maayanlab.cloud/Harmonizome/gene_set/cholangiocyte/TISSUES+Text-mining+Tissue+Protein+Expression+Evidence+Scores; last visited on 21 August 2025), which consists of genes specific to bile duct-lining epithelial cells (i.e., cholangiocytes).

**Figure 7:**
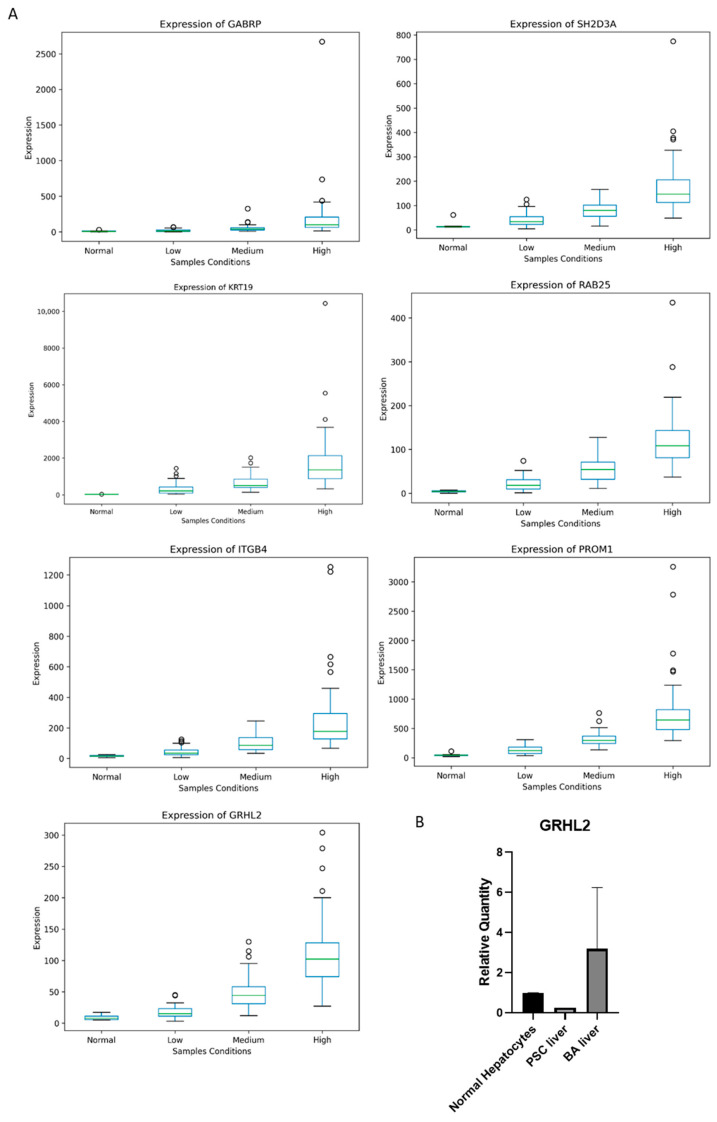
Expression plots of *ESRP1* co-expressed genes in BA. (**A**) Genes showing similar trend in expression with ESRP1 in BA datasets. (**A**) Genes that exhibit a similar trend in expression to *ESRP1* in BA datasets were identified through bioinformatic analysis. Representative genes with a high correlation to *ESRP1* expression are displayed. *GABRP*, *SH2D3A*, *KRT19*, *RAB25*, *ITGB4*, *PROM1* and *GRHL2* are part of the cholangiocyte gene set (Harmonizome 3.0 database accessed on 21 October 2025). (**B**) qRT-PCR validation of *GRHL2* expression was conducted in human normal hepatocytes (*n* = 1) and liver samples from PSC (*n* = 1) and BA (*n* = 2) subjects.

This analysis identified twelve shared genes, *AQP1*, *CFTR*, *GABRP*, *GRHL2*, *ITGB4*, *KRT19*, *KRT7*, *PROM1*, *RAB25*, *SCTR*, *SH2D3A*, and *THY1*, highlighting a subset of *ESRP1*-associated transcripts with strong cholangiocyte/ductal epithelial relevance (some of these are shown in Figure 7).

To further refine this selection, we evaluated the differential expression and correlation (with ESRP1) of these seven genes in the two contexts where ESRP1 alterations were observed: (i) CCA versus normal tissues (CHOL TCGA), and (ii) cholangiocyte cell lines representing normal (NHC_WCE), immortalized (H69_WCE), and tumor-derived (EGI_WCE, TFK_WCE) states. Interestingly, seven genes (GABRP, GRHL2, ITGB4, KRT19, PROM1, RAB25 and SH2D3A) exhibited statistically significant upregulation under disease conditions (Figure S8) and showed a positive and significant correlation with ESRP1 expression.

## 4. Discussion

The early and accurate diagnosis of cholangiopathies remains challenging due to overlapping clinical features and the lack of specific, non-invasive biomarkers, which often leads to late-stage detection and limited treatment options [49,50,51]. Identifying molecular markers capable of distinguishing among disorders of the biliary epithelia is therefore crucial, as these markers can enable clinicians to stratify patients for timely and targeted therapies, improving prognosis and supporting personalized treatment strategies. Incorporating molecular markers into routine diagnostics not only facilitates early detection but also helps identify patients who may benefit from specific targeted treatments or clinical trials, ultimately enhancing survival and quality of life [51,52].

Our results demonstrate for the first time that ESRP1 is expressed in the livers of mice with cholestatic injury, fibrosis, and prominent ductular reaction. Notably, ESRP1 expression is slightly upregulated as early as Day 1 post-BDL, suggesting that it is induced during early stages of injury, coinciding with hepatic stem cell (HSC) and biliary epithelial cell (BEC) activation, before the onset of detectable liver fibrosis (Figure 2) [53]. This time point correlates with the overexpression of alpha-smooth muscle actin (α-SMA, a marker of hepatic stellate cell activation), transforming growth factor-β2 (TGF-β2) and S100a4 (a marker associated with cells undergoing EMT and involved in fibrogenesis) in the BDL model. Similarly, BECs from BA exhibit high levels of α-SMA and S100a4, as well as TGFβ2 compared with normal livers, showing the potential of ESRP1 as an early marker/regulator of EMT in obstructive cholangiopathies [36,54]. ESRP1 is also highly expressed in the livers of patients with BA and CFLD but barely discernible in PSC and PBC. A short isoform, approximately 50 kDa, was also observed in the livers, confirming the presence of alternatively spliced ESRP1 isoforms, as previously reported in other tissues, and warrants further studies at the functional level [55,56]. PSC and PBC are both immune-mediated cholestatic liver diseases, but they differ clinically and molecularly from BA and CFLD. Clinically, PSC is characterized by chronic inflammation, bile duct destruction, and progressive fibrosis, often accompanied by periductal scarring and a strong association with inflammatory bowel disease, while PBC features chronic cholangitis, bile duct loss, and classical autoimmune markers; both conditions can progress to cirrhosis and liver failure [57,58]. The ductopenia or loss/absence of interlobular bile ducts in portal tracts, which is a key histological feature in both PBC and PSC, likely explain the absence of ESRP1 expression in these diseases [57,59].

The DDC-treated mouse model provides a useful approximation of PSC; however, certain molecular features, including ESRP1 regulation, may not be fully recapitulated, further supporting the reported differences between the mouse model and livers of PSC patients [60]. On the other hand, both BA and CFLD are characterized by a prominent ductular reaction. In BA, the ductular reaction is a hallmark hepatic pathology, characterized by a marked expansion of biliary epithelial cells within the portal area and associated with biliary reprogramming, fibrosis, and inflammation [61,62]. The ductular reaction in BA involves the upregulation of specific biliary markers and transcription factors, contributing to disease progression and liver injury [61,62]. In CFLD, a bile acid-induced ductular reaction is also observed, where taurocholate promotes liver progenitor cell proliferation and biliary differentiation, ultimately leading to the development of the ductular reaction, hepatic stellate cell recruitment and fibrosis [63]. The extent of this ductular reaction in CFLD correlates with both the stage of hepatic fibrosis and bile acid levels, indicating its central role in disease pathogenesis. Both BA and CFLD are thus classified as obstructive cholangiopathies which, according to our data, exhibit high hepatic ESRP1 expression. These findings suggest that ESRP1 should be further investigated in a larger cohort of cholangiopathic patients with diverse etiologies to validate its potential as a molecular marker capable of distinguishing obstructive biliary diseases from immune-mediated forms.

The generation and analysis of CHOs from BA patients provide a valuable ex vivo model to study disease-specific cellular and molecular features, including ESRP1 expression. The morphological differences observed in BA-CHOs, such as discontinuities in the outer capsule and altered transporter function, reflect pathological changes seen in BA livers. Notably, the upregulation of both epithelial (CDH1) and mesenchymal (Vimentin) markers, suggests a partial EMT and highly plastic cellular states characteristic of BA pathology. Furthermore, the induction of ESRP1 in healthy CHOs following BLZ treatment, which recapitulates BA-like features, supports the idea that ESRP1 upregulation is a response to cholangiocyte injury and may be linked to disease onset [42]. The temporal correlation between ESRP1 upregulation and the emergence of BA-like morphological changes in BLZ-treated organoids further implicates ESRP1 as a marker of disease-specific epithelial remodeling. These findings highlight the potential of ESRP1 not only as a diagnostic biomarker for BA but also as a molecular participant in the pathophysiological processes underlying cholangiocyte dysfunction and repair. Organoid models thus offer a powerful platform for dissecting the mechanistic role of ESRP1 and for testing targeted interventions in BA and related cholangiopathies [64].

Mechanistically, ESRP1 is a key regulator of alternative splicing and EMT, processes that are central to tissue remodeling, fibrosis, and cancer progression [29,65]. In intrahepatic CCA and other cancers, ESRP1 modulates tumor progression and metastasis by interacting with EMT-related factors such as ZEB1 and by regulating the splicing of genes like CD44 and LRRFIP2, which influence cellular phenotype and metastatic potential. Although this study focuses on non-malignant cholangiopathies, the upregulation of ESRP1 in ductular reactions and progenitor cell expansion suggests that similar splicing and EMT-related mechanisms may operate in chronic biliary injury. This raises the possibility that ESRP1 not only serves as disease marker but may actively contribute to cholangiopathy pathophysiology by modulating cholangiocyte plasticity and the fibrogenic response. Interestingly, further examination of our previously extracted datasets revealed that ESRP1, as well as the five cholangiocyte-derived ESRP1-related genes, are statistically significantly upregulated in CCA tumor tissues [6]. This finding suggests that ESRP1 expression may correlate with the severity of cholangiopathies, particularly when pathological changes approach tumorigenesis, as in PSC- to -CCA progression. Therefore, ESRP1 expression in the hepatobiliary system may be an indicator of severe pathogenicity in patients. However, the precise functional role of ESRP1 in biliary disorders remains to be elucidated, and additional mechanistic studies are needed to establish whether ESRP1 acts as a driver or a bystander in disease progression.

Our bioinformatics data provide insights into the pathways that should be investigated in future studies to understand the mechanistic and clinical relevance of ESRP1. In BA, ESRP1 expression is significantly upregulated in liver tissues compared to normal controls, indicating its involvement in the disease pathogenesis [66]. Functional enrichment of genes co-expressed with ESRP1 in the ESRP1high subgroup highlights the activation of inflammatory, immune, and metabolic pathways, including chemotaxis, leukocyte migration, oxidative and endoplasmic reticulum stress responses, and wound healing, reflecting a reactive hepatic microenvironment. Cellular components and molecular functions enriched include extracellular matrix remodeling, focal adhesion, integrin and collagen binding, and metallopeptidase activity, which are consistent with tissue inflammation and fibrogenesis. KEGG pathway analysis further implicates cytokine-cytokine receptor interaction, NF-κB signaling, T cell receptor signaling, and fibrogenic pathways such as TGF-β and Hedgehog signaling, suggesting that immune activation and fibrosis contribute to BA pathogenesis.

Correlation analyses further identify ESRP1-associated genes involved in epithelial organization, extracellular matrix reorganization, EMT, and TGF-β response, indicating a potential role of ESRP1 in promoting ductal epithelial resilience and controlled fibrogenic remodeling. Overall, ESRP1-related pathways in BA regulate immune infiltration, oxidative stress, matrix remodeling, and metabolic adaptation, collectively driving the pathogenic process in BA. Notably, ESRP1 and GRHL2 show parallel upregulation in BA, suggesting a coordinated role in maintaining or reprogramming the epithelial phenotype during cholangiocyte injury and repair. ESRP1 regulates alternative splicing of EMT-related genes, whereas GRHL2 functions as a transcription factor that promotes epithelial gene expression and suppressing EMT [67,68]. Their co-expression may represent a protective or compensatory mechanism to preserve epithelial characteristics, counteracting fibrogenic or mesenchymal transitions that drive disease progression, and supporting ductular reaction, progenitor cell expansion, and epithelial repair. This ESRP1-GRHL2 axis may thus represent a potential target to modulate epithelial plasticity and influence BA outcomes, warranting further mechanistic studies to clarify their functional interplay in cholangiopathies.

Several biomarkers for BA have shown strong clinical potential, but only a few are currently integrated into routine clinical practice. Matrix metalloproteinase-7 (MMP-7) is the most validated and widely used biomarker, exhibiting high sensitivity and specificity for distinguishing BA from other causes of neonatal cholestasis. Its diagnostic accuracy is further enhanced when combined with γ-glutamyl transferase (GGT) and it is increasingly incorporated into diagnostic algorithms [69,70,71,72]. Serum microfibril-associated glycoprotein 4 (MFAP4) is another marker that accurately reflects liver fibrosis and prognosis in BA; however, its clinical use remains limited and is not yet a standard practice in most centers [73]. The aspartate aminotransferase-to-platelet ratio index (APRi) is a non-invasive tool for assessing liver fibrosis and cirrhosis, but its clinical utility remains unclear due to variability in study results and lack of standardized cut-off values [74].

Other promising markers, including polymeric immunoglobulin receptor (PIGR), LOX-1+ PMN-MDSCs, and ANGPTL6, have demonstrated diagnostic or prognostic value in recent studies, but are not yet routinely used in clinical practice and require further validation [75,76,77]. In summary, while MMP-7, often in combination with GGT, is now being used clinically for BA diagnosis, most other biomarkers remain in the research or early clinical validation phase, leaving room for biomarker discovery, particularly when combined with other markers, could improve specificity and sensitivity in distinguishing among cholangiopathies.

This study has some limitations, including the relatively small patient sample size, which is inherent to these for these rare diseases and the need for validation in larger, more diverse cohorts. Future research should aim to delineate the downstream targets of ESRP1 in cholangiocytes, exploring its role in EMT and fibrosis, and evaluating its potential as a therapeutic target or clinical biomarker. Nevertheless, our findings are reinforced by bioinformatics analyses conducted on large patient cohorts, providing a robust foundation for investigating ESRP1 expression and function in obstructive cholangiopathies.

## 5. Conclusions

Our findings highlight the critical need for further investigation into the mechanisms driving the overexpression of ESRP1 and other related post-transcriptional regulators in cholangiopathies. Such research may reveal novel biomarkers and therapeutic strategies for the effective management of debilitating biliary diseases. By elucidating the role of ESRP1 in epithelial plasticity, EMT, and fibrosis, future studies could pave the way for improved diagnostic tools, patient stratification, and targeted interventions in obstructive and immune-mediated biliary disorders.

## Figures and Tables

**Table 1:** Characteristics of BA and PSC patients who underwent liver biopsy (used for CHO generation).

A.	Patient	Age at Biopsy/Years	Sex	Disease Diagnosed	KPE	LT
	BA1	0.15	M	BA	Yes	No
	BA2	0.16	F	BA	Yes	Yes
	PSC	7.16	F	PSC + AIH	No	No

Abbreviations: AIH, autoimmune hepatitis; BA, biliary atresia; KPE, Kasai portoenterostomy; LT, liver transplantation; PSC, primary sclerosing cholangitis.

## Data Availability

The original contributions presented in this study are included in the article/Supplementary Materials. Further inquiries can be directed to the corresponding author.

## Supplementary Materials

The following supporting information can be downloaded at https://www.mdpi.com/article/10.3390/biom16010009/s1, Figure S1: ESRP1 expression in livers of mice fed a DDC diet; Figure S2: Molecular profiling of BA patients’ livers. Livers from a healthy donor (HD) and two BA patients (BA) Quantitative real time-PCR data are shown; Figure S3: Characterization of CHOs; Figure S4: BLZ treatment of CHOs. Cell size analysis revealed that healthy control CHO cells exhibited an increase in size over time in culture. In contrast, CHO cells exposed to BLZ showed a reduction in cell size in both a dose-dependent and time-dependent manner. The cytotoxic effects were most pronounced at a BLZ concentration of 20 µM versus 2 µM; Figure S5: ESRP1 expression profiles across different conditions; Figure S6: *GO* analysis of differentially expressed genes (DEGs) in the ESRP1^low^ group with respect to normal subjects. The top enriched categories for (i) Biological Process (BP), (ii) Cellular Component (CC), (iii) Molecular Function (MF), and (iv) KEGG pathway analysis are shown. Related to Figure 5 in the text; Figure S7: GO of genes positively correlated with ESRP1 in the ESRP1low group; Figure S8: Expression profiles across different conditions of GABRP, GRHL2, KRT19, RAB25, SH2D3A, ITGB4 and PROM1l; Figure S9: Uncropped western blot images from Figure 3C; Table S1: qRT-PCR Primers used in this study; Table S2: GO and KEGG significant enrichments for the comparisons discussed in the paper belonging to the following analysis: Differential Expression (DE) in ESRP1high and ESRP1low BA samples compared to Normal Subjects; Correlation (CORR) in all BA samples and ESRP1high and ESRP1low respectively.

## Abbreviations

The following abbreviations are used in this manuscript:

ABCC1	ATP-binding cassette, sub-family C (CFTR/MRP), member 1
ABCC3	ATP-binding cassette, sub-family C (CFTR/MRP), member 3
APRi	Aspartate aminotransferase-to-platelet ratio index
BA	Biliary Atresia
BA-CHOs	Biliary Atresia-Cholangoids
BDL	Bile Duct Ligation
BLZ	Biliatresone
BP	Biological Process
CC	Cellular Component
CFLD	Cystic Fibrosis-related liver disease
CHO(s)	Human Cholangiocyte Organoid(s)
CK7	Cytokeratin-7
CK19	Cytokeratin-19
CCl_4_	Carbon Tetrachloride
CYP	Cytochrome
DDC	Diethoxycarboncyl-1,4-dihydrocollidine
DE	Differential expression
DEG	Differentially Expressed Genes
EMT	Epithelial-to-mesenchymal Transition
ESRP	Epithelial Splicing Regulatory Protein
FXR	Farnesoid X receptor
GEO	Gene Expression Omnibus
GGT	γ-glutamyl transferase
GO	Gene Ontology
H-CHOs	Healthy-Cholangoids
H&E	Hematoxylin and Eosin
HNF1B	HNF1 homeobox B
KEGG	Kyoto Encyclopedia of Genes and Genomes
KPE	Kasai portoenterostomy
Mdr	Multiple drug resistance
MF	Molecular Function
MFAP4	Microfibril-associated glycoprotein 4
PBC	Primary Biliary Cholangitis
PHx	Partial Hepatectomy
PIGR	polymeric immunoglobulin receptor
PSC	Primary Sclerosing Cholangitis
RBP(s)	RNA-binding protein(s)
Rho123	Rhodamine 123
TGF-β	Transforming growth factor-β
ZO-1	Zona occludens 1
