# Supplementary material for: Elevated Epithelial Splicing Regulatory Protein 1 Expression in Biliary Atresia Indicates Its Potential as a Molecular Marker
Source: Biomolecules. 2025 Dec 19;16(1):9. doi: 10.3390/biom16010009 (PMC12838563; doi:10.3390/biom16010009)
Supplement: Supplementary file 1 [file biomolecules-16-00009-s001.zip › Table S1.pdf]

Table S1: qRT-PCR Primers used in this study.

qRT-PCR primers (SYBR)			
**Primer name**	**Forward (5′-3′)**	**Reverse (5′-3′)**	**Reference**
ESRP1	GCTTATCCCCACCGCCAT	GCAGTGCTCGTGGATTCAAA	NIH PRIMER DESIGNING TOOL (https://www.ncbi.nlm.nih.gov/tools/primer-blast/; last visited on 15/03/2024)
CDH1	GCATTGCCACATACACTCTCTTCT	GCTTGTTGTCATTCTGATCGGTTA	
VIMENTIN	CCTGTGAAGTGGATGCCCTTA	AGCTTCAACGGCAAAGTTCTCT	
CK7	GACATCGAGATCGCCACCTAC	GATATTCACGGCTCCCACTCC	
SOX9	GAAGGACCACCCGGATTACA	CCTTGAAGATGGCGTTGGG	
GGT-1	CAGAGATTGCCTGGAACACCA	CACCTCACTTGCCTTGCTGA	
CK-19	GAGCTGGCCTACCTGAAGAAGA	TTGGCTTCGCATGTCACTCA	
ZO-1	CGAGTTGCAATGGTTAACGGA	TCAGGATCAGGACGACTTACTGG	
HNF1B	TCACAGATACCAGCAGCATCAGT	GGGCATCACCAGGCTTGTA	
GRHL2	CGCCTATCTCAAAGACGACCAG	CCAGGGTGTACTGAAATGTGCC	https://www.origene.com/catalog/gene-expression/qpcr-primer-pairs/hp215126-grhl2-human-qpcr-primer-pair-nm-024915 (last visited on 20/10/2025)
