# Supplementary figures and images for: Elevated Epithelial Splicing Regulatory Protein 1 Expression in Biliary Atresia Indicates Its Potential as a Molecular Marker
Source: Biomolecules. 2025 Dec 19;16(1):9. doi: 10.3390/biom16010009 (PMC12838563; doi:10.3390/biom16010009)

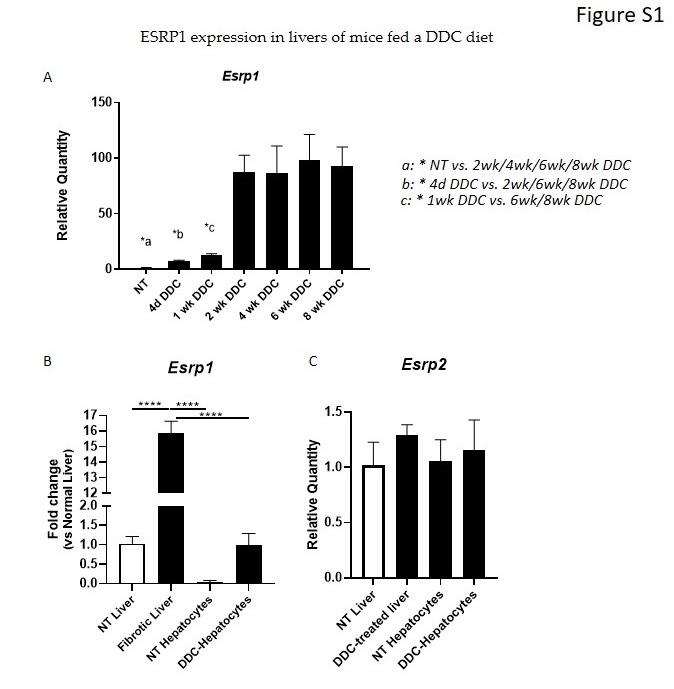

Supplement: Supplementary file 1 [file biomolecules-16-00009-s001.zip › Figure S1.JPG]

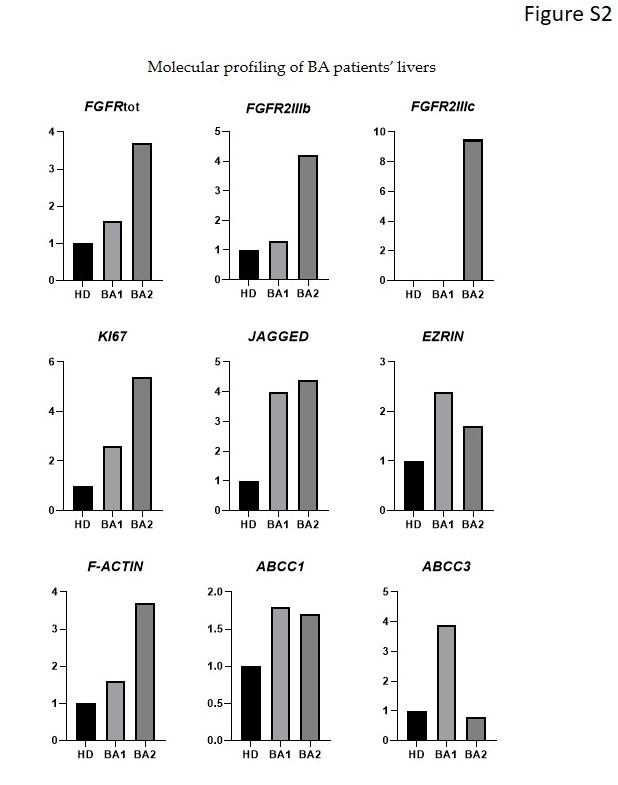

Supplement: Supplementary file 1 [file biomolecules-16-00009-s001.zip › Figure S2.JPG]

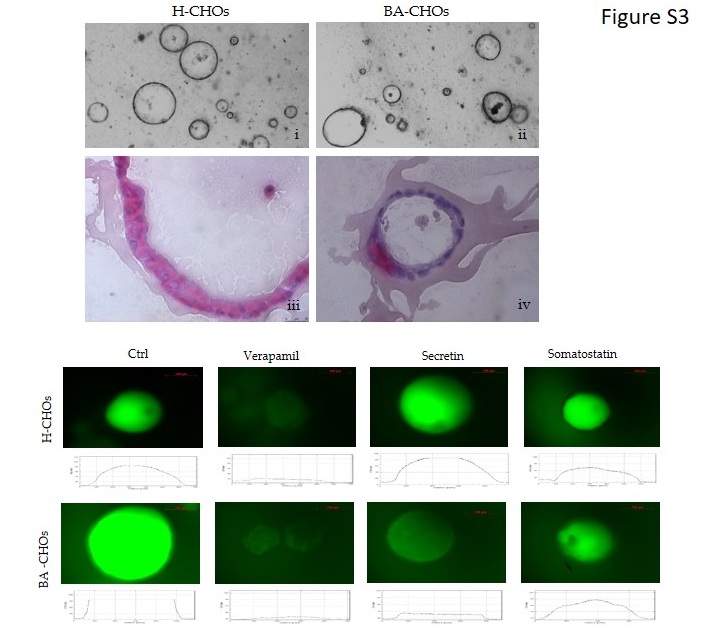

Supplement: Supplementary file 1 [file biomolecules-16-00009-s001.zip › Figure S3.JPG]

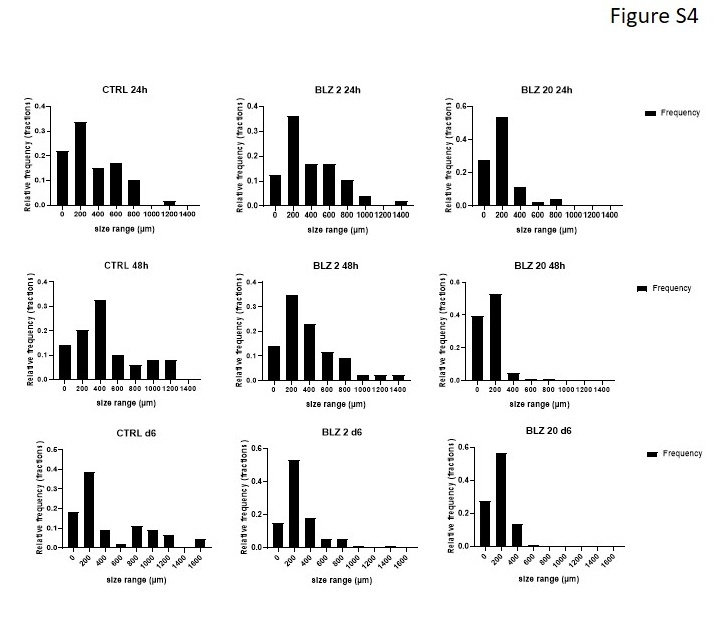

Supplement: Supplementary file 1 [file biomolecules-16-00009-s001.zip › Figure S4.JPG]

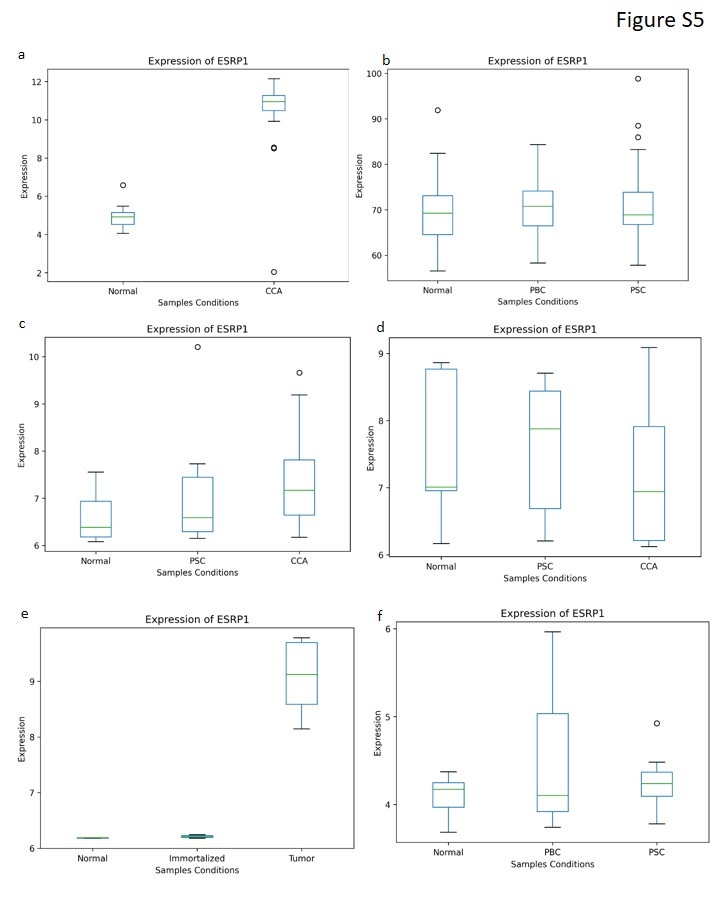

Supplement: Supplementary file 1 [file biomolecules-16-00009-s001.zip › Figure S5.JPG]

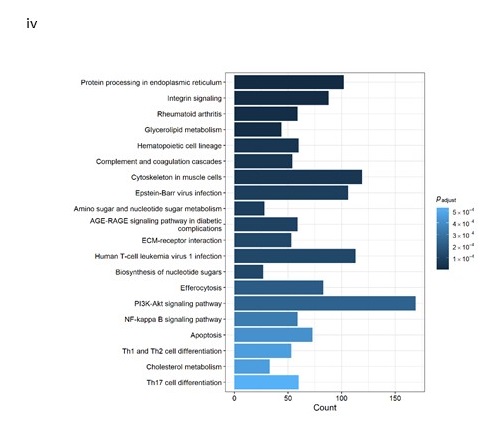

Supplement: Supplementary file 1 [file biomolecules-16-00009-s001.zip › Figure S6 cont.JPG]

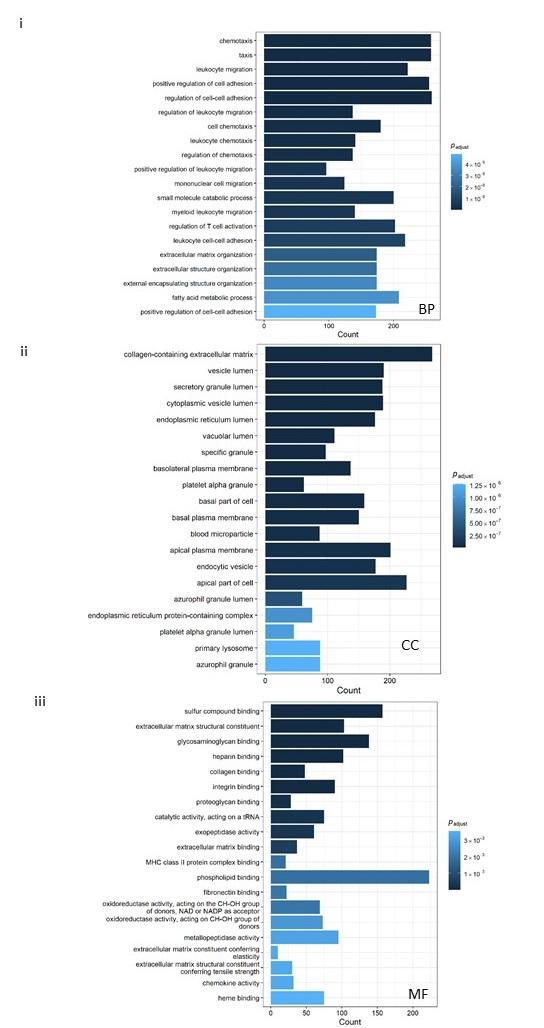

Supplement: Supplementary file 1 [file biomolecules-16-00009-s001.zip › Figure S6.JPG]

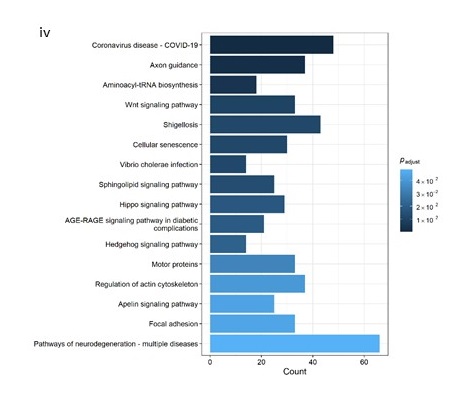

Supplement: Supplementary file 1 [file biomolecules-16-00009-s001.zip › Figure S7 cont.JPG]

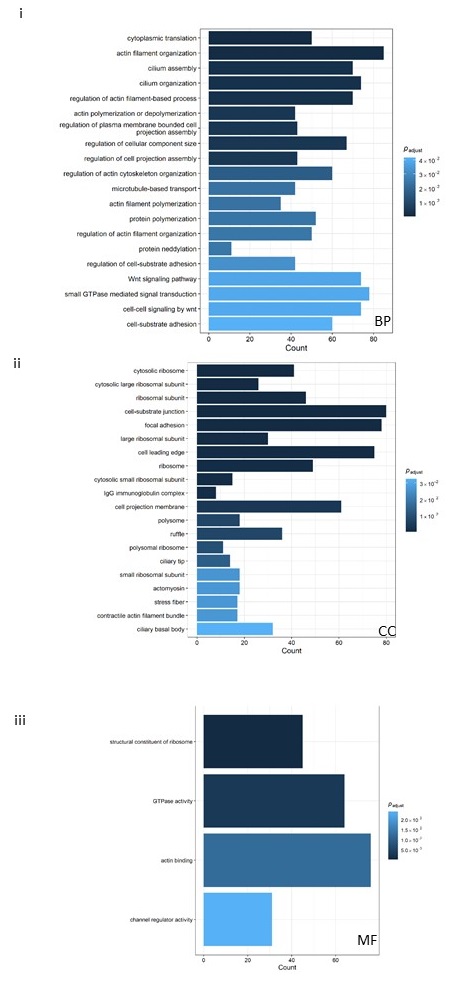

Supplement: Supplementary file 1 [file biomolecules-16-00009-s001.zip › Figure S7.JPG]

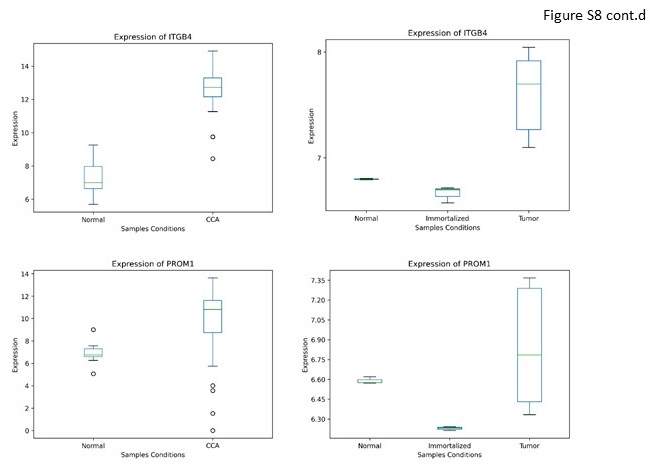

Supplement: Supplementary file 1 [file biomolecules-16-00009-s001.zip › Figure S8 cont.jpg]

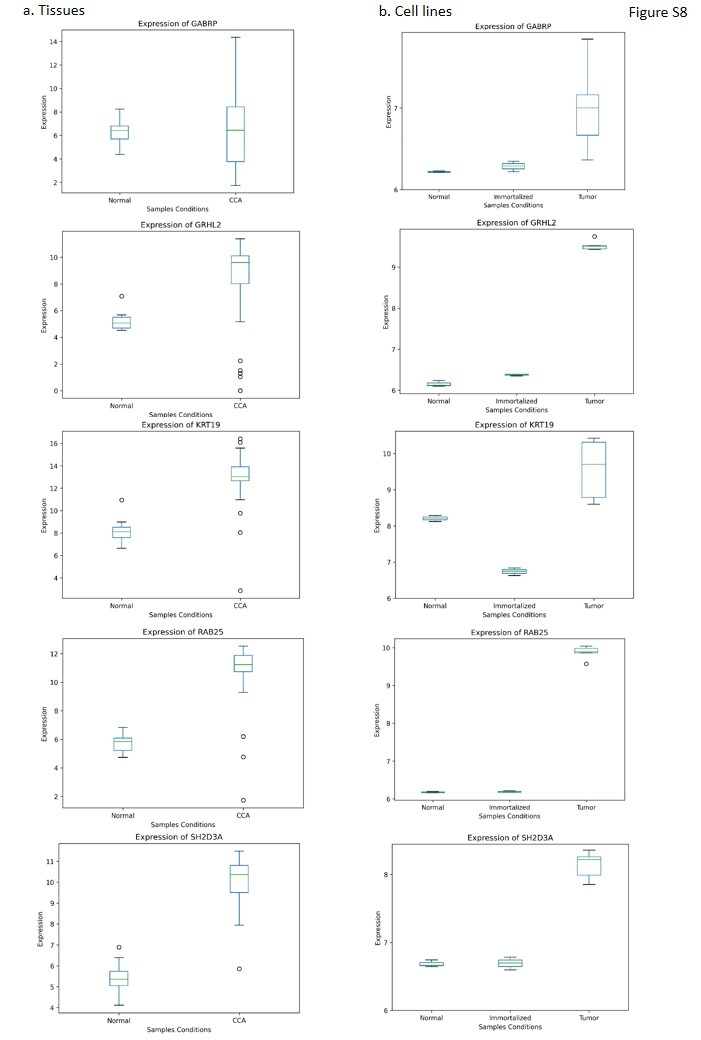

Supplement: Supplementary file 1 [file biomolecules-16-00009-s001.zip › Figure S8.JPG]

# Western blot with anti-ESRP1 (HPA023719\_Atlas) and anti-vinculin antibodies

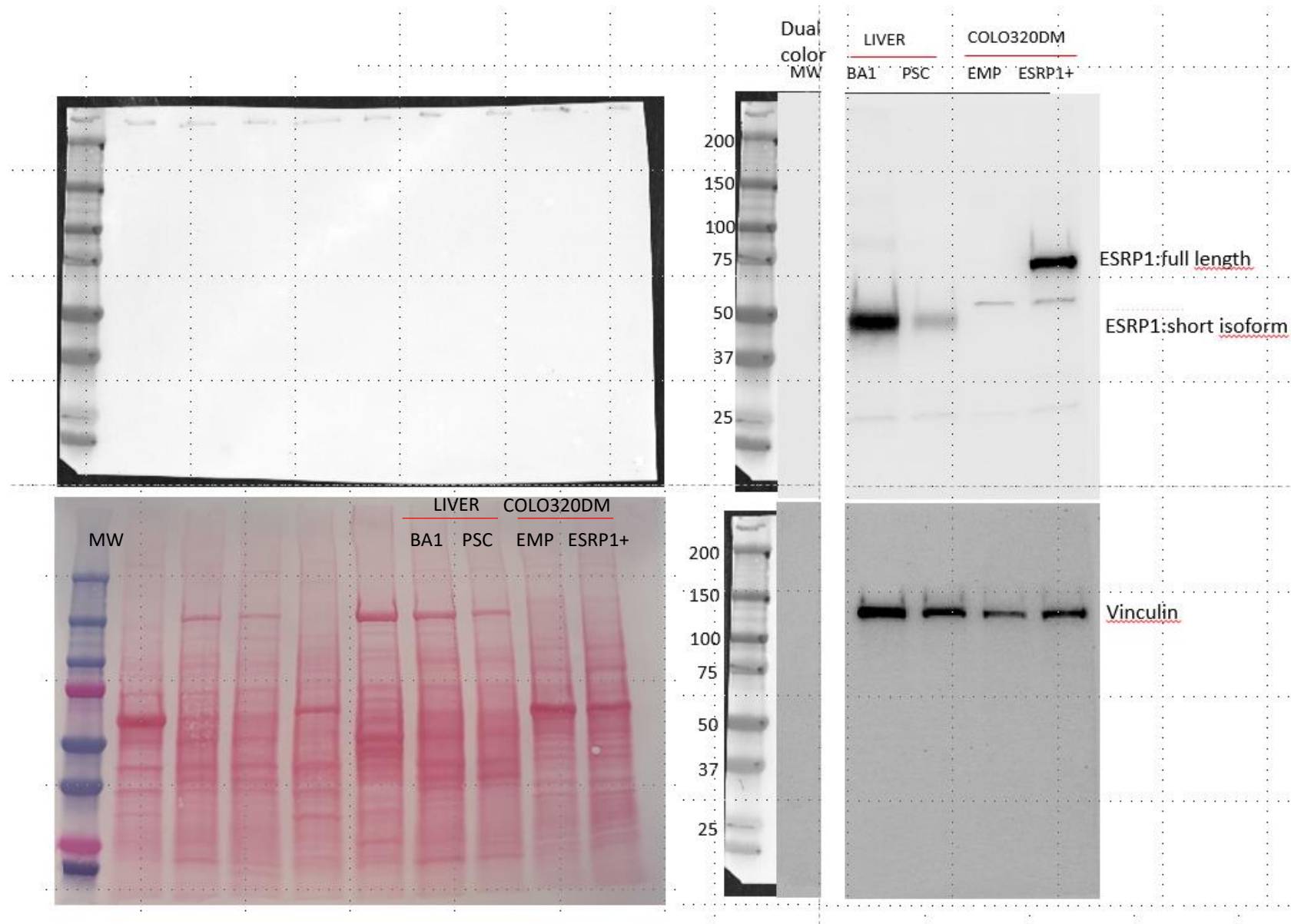

Supplement: Supplementary file 1 [file biomolecules-16-00009-s001.zip › Figure S9.pdf]
